# Risk stratification for CMV reactivation in sepsis patients: development of an interpretable machine learning model

**DOI:** 10.1186/s12879-025-12154-0

**Published:** 2025-12-22

**Authors:** Chengcheng Shen, Yang Chen, Tingting Pan, Hongping Qu, Ling Zhang, Rui Tian, Tong Wu, Ruoming Tan

**Affiliations:** 1 Department of Critical Care Medicine, Ruijin Hospital, Shanghai Jiao Tong University School of Medicine, 197 Ruijin Er Road, Shanghai, 200025 China; 2 College of Health Science and Technology, Shanghai Jiao Tong University School of Medicine, Shanghai, 200025 China

**Keywords:** CMV, Reactivation, Sepsis, ICU, Interpretable machine learning, SHAP, APACHE II score, CD4^+^ lymphocyte count, Corticosteroid use, Risk stratification

## Abstract

**Background:**

Cytomegalovirus (CMV) reactivation in critically ill populations is associated with adverse outcomes. This study aimed to develop and validate an interpretable machine learning model to predict CMV reactivation within 28 days of ICU admission in sepsis patients, providing a risk stratification tool to optimize clinical management.

**Methods:**

A retrospective cohort study was conducted using data from intensive care unit (ICU) patients meeting SEPSIS-3 criteria. Key clinical variables, including acute physiology and chronic health evaluation Ⅱ(APACHE Ⅱscore), CMV IgG titer, CD4^+^ lymphocyte count, and corticosteroid use duration, were selected through univariate logistic regression. Seven machine learning models were developed, with the gradient boosting machine (GBM) demonstrating superior performance. Model interpretability was enhanced using Shapley Additive Explanations (SHAP) to identify critical predictors and visualize feature contributions.

**Results:**

Among 221 patients, 19.0% experienced CMV reactivation. The test set AUC values for GBM, logistic regression (LR), neural network (NN), support vector machine (SVM), random forest (RF), adaptive boosting (AdaBoost) and k-nearest neighbors (KNN) models were recorded as 0.761, 0.684, 0.673, 0.659, 0.65, 0.624, and 0.623, respectively. GBM model achieved the highest AUC in the test set, with robust calibration and net benefit of the GBM model in the test set exceeded that of the other models at threshold probabilities ranging from 10% to 60%. SHAP analysis identified the APACHE II score, corticosteroid use, CMV IgG levels, and CD4 + lymphocyte count as the top four important predictors. Kaplan-Meier curves demonstrated significant stratification between high- and low-risk groups (*p* < .05).

**Conclusion:**

This interpretable machine learning model provides accurate CMV reactivation prediction and clear insights into contributing factors. It has the potential to enhance early risk stratification and guide targeted management strategies for sepsis patients in ICU settings.

**Supplementary Information:**

The online version contains supplementary material available at 10.1186/s12879-025-12154-0.

## Background

Cytomegalovirus (CMV) reactivation is a common complication in critically ill patients, particularly those with sepsis, and is associated with adverse clinical outcomes. CMV, a β-herpesvirus that establishes lifelong latency, can reactivate when the host’s immune system is compromised. This reactivation occurs frequently in intensive care unit (ICU) patients, with some studies reporting rates as high as 71% [1]. In septic patients, CMV reactivation has been linked to worse clinical outcomes, including increased mortality, prolonged ICU and hospital stays, extended mechanical ventilation duration, and a higher risk of secondary infections [2–5]. These associations underscore the importance of early identification of high-risk patients, which can facilitate prompt detection of CMV reactivation and guide antiviral therapy, potentially improving clinical outcomes.

Identifying patients at high risk for CMV reactivation is challenging due to inconsistent risk factors reported across studies. For instance, while some studies identify the severity of illness as a significant risk factor [2, 3], others fail to demonstrate such an association [4, 6]. Traditional detection methods, such as PCR, antigen testing, and viral culture of blood or other bodily fluids, are commonly used to diagnose CMV infection [5, 7, 8]. Detection methods based on evaluating CMV-specific immune responses often mean that infection has already occurred when the results are positive. These methods all have certain delays in detecting the infection. Eleftheria Kampouri [9] used interferon-gamma (IFN-γ) release assays to quantify the strength of CMV-specific cell-mediated immunity as a predictive factor for CMV reactivation in patients undergoing CART therapy. Alda Saldan [10] employed cytomegalovirus enzyme-linked immunospot assays to predict congenital CMV infection. Although these methods are effective, some tests are time-consuming, costly, and difficult to implement widely, limiting their utility in early clinical decision-making.

Machine learning (ML) has shown significant potential as a diagnostic and risk assessment tool in healthcare [11]. ML models have been applied to predict CMV reactivation in various patient populations, such as hematopoietic stem cell transplant recipients, using both baseline and time-dependent laboratory data [12]. However, reliable and efficient ML models specifically designed to predict CMV reactivation in sepsis patients are still lacking. A common limitation of ML models is their “black box” nature, where the complex and opaque decision-making processes hinder clinical adoption and limit interpretability. To address this challenge, interpretability techniques like SHapley Additive exPlanations (SHAP), based on game theory, have been developed to elucidate feature contributions to model predictions [10]. SHAP assigns a score to each feature based on its contribution on the model’s output, enabling both local and global interpretability, thereby enhancing the transparency and trustworthiness of machine learning models [13].

In this study, we aim to develop an ML model using retrospective data to predict CMV reactivation within 28 days in sepsis patients. To enhance interpretability, SHAP analysis will be utilized, enabling effective risk stratification and the early identification of high-risk individuals. Our goal is to facilitate timely interventions, optimize patient management, and ultimately improve outcomes for critically ill patients at risk of CMV reactivation.

## Methods

### Study population

**Fig. 1:**
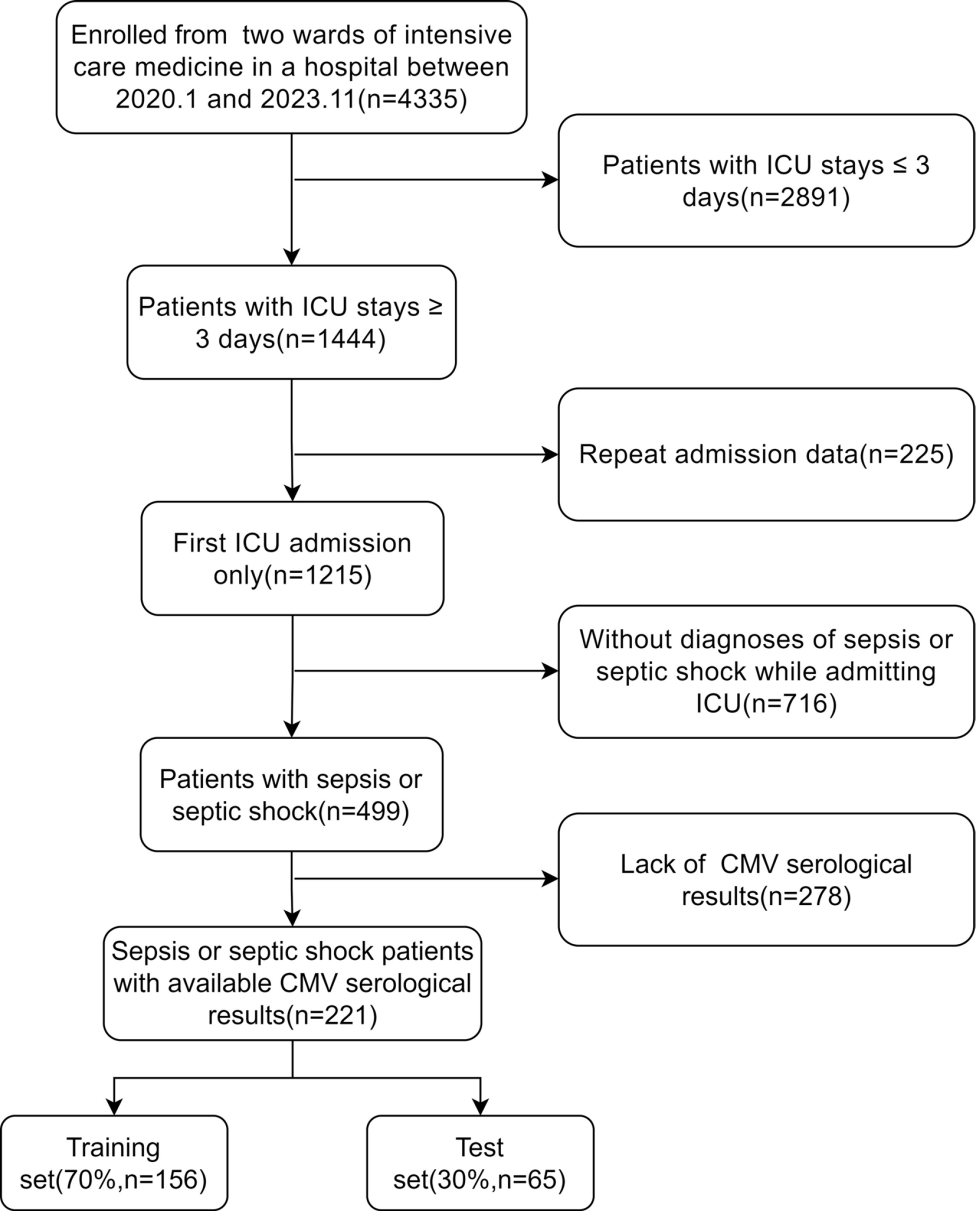
Flowchart of patient selection. *Abbreviations*: ICU, intensive care unit; CMV, cytomegalovirus

We retrospectively collected data from two ICU departments at Ruijin Hospital, affiliated with Shanghai Jiao Tong University School of Medicine, between January 2020 and November 2023. The study was approved by Ruijin Hospital, Shanghai Jiao Tong University School of Medicine. Figure 1 illustrates the patient inclusion process. Inclusion criteria were as follows: (1) Age ≥ 18 years; (2) ICU stay duration ≥ 3 days; (3) Diagnosis meeting SEPSIS-3 criteria upon ICU admission; (4) Availability of CMV IgM or CMV DNA test results every two weeks after ICU admission; (5) CMV IgG test result available either prior to or within 3 days of ICU admission; (6) First ICU admission. The ICU stay is defined as the time interval from the electronic health records-recorded ICU admission timestamp to the ICU discharge timestamp. For inter-ICU transfers occurring within the same hospitalization, we consolidated multiple transfers into a single continuous stay. Only the first ICU admission during the index hospitalization was included in the analysis. Sepsis was diagnosed according to the SEPSIS-3 criteria, which defines sepsis as an increase in the Sequential Organ Failure Assessment (SOFA) score(Supplementary Table 1) of ≥ 2 points due to infection [14]. Exclusion criteria were as follows: (1) Pregnant or breastfeeding women; (2) Missing key clinical data or laboratory test results; (3) End-stage cancer or other terminal diseases; (4) HIV/AIDS infection; (5) Discontinue treatment measures.

### Data collection

Data collected included patient age, sex, body mass index (BMI), and the highest APACHE II score within 24 h of ICU admission. Other variables included infection site, comorbidities (including diabetes, coronary atherosclerotic heart disease, chronic kidney disease, autoimmune diseases, and history of malignancy), CMV IgG titer within 3 days of ICU admission, CMV IgM titer within 28 days, serum CMV DNA copy number within 28 days, lymphocyte count, platelet count, neutrophil count, C-reactive protein, alanine transaminase(ALT), aspartate transaminase(AST), CD3^+^ lymphocyte count, CD4^+^ lymphocyte count, CD8^+^ lymphocyte count, activated partial thromboplastin time (APTT), prothrombin time (PT), D-dimer, procalcitonin (PCT), and prealbumin within 1 day of ICU admission. Additionally, the following data were recorded: the number of days on mechanical ventilation, duration of intravenous corticosteroid use, duration of vasoactive agent use within 28 days of ICU admission, and 90-day mortality.

### Study outcomes

The primary outcome of this study was the prediction of a binary event: the occurrence of CMV reactivation within 28 days of ICU admission. In this study, CMV reactivation was defined as the first instance of a CMV IgM titer or CMV DNA copy number exceeding the threshold (CMV IgM titer: ≥1.00 AU/ml, CMV DNA copy number: ≥250 IU/ml) [15].

### Feature selection and model development

We stratified the study population based on outcome and divided it into a training set and a test set in a 7:3 ratio. In the training set, univariate logistic regression was performed with CMV serostatus (positive within 28 days) as the dependent variable. Variables with a p-value ≤ 0.05 were selected as predictive features for training the machine learning model. To evaluate model performance in the training set, a 10-fold cross-validation with repetition was applied. Seven machine learning models were developed and trained: logistic regression (LG), support vector machines (SVM), gradient boosting machines (GBM), neural networks (NN), random forests (RF), k-nearest neighbors (KNN), and adaptive boosting (AdaBoost). The hyperparameters used in each model are summarized in Supplementary Table 2.

### Model evaluation

Model performance was evaluated using the area under the receiver operating characteristic (ROC) curve (AUC). After applying the models to the training set, the model with the highest AUC was selected for subsequent SHAP analysis. Multiple evaluation metrics, including accuracy, sensitivity, specificity, precision, F1 score, and AUC, were used to comprehensively assess the discriminative ability of the machine learning models. Calibration curves were generated to evaluate model calibration, and clinical decision curve analysis (DCA) was performed to assess clinical benefit. CIs for model performance metrics were calculated as follows. For the AUC of ROC, 95% CIs were calculated using DeLong’s method. For other performance metrics, 95% CIs were estimated using non-parametric bootstrapping with 1,000 resamples. The optimal probability threshold, determined using the Youden Index, was applied to stratify patients into low- and high-risk groups. Kaplan-Meier curves were compared between these groups using the Log-rank test to evaluate differences in incidence of CMV reactivation.

### Model interpretation and feature importance

Machine learning models are often considered “black boxes”. To address this, we applied the SHAP technique [13, 16], which provides both local and global interpretability. SHAP was used to interpret the best-performing model, revealing the importance of each feature and enhancing the model’s interpretability and credibility. Additionally, we presented the prediction results for one positive and one negative case from the training set.

### Statistical analysis

All statistical analyses were performed using R (version 4.2.2); a list of the main packages used is provided in Supplementary Table 3. For continuous variables, normality was assessed using the Shapiro–Wilk test. For normally distributed variables with homogeneity of variance, data are presented as mean ± standard deviation (SD). Group comparisons (between patients with and without CMV reactivation) were conducted using the independent samples t-test, which tests the null hypothesis that the population means of the two groups are equal (H₀: µ₁ = µ₂) against the two-sided alternative (H₁: µ₁ ≠ µ₂). For non-normally distributed variables, data are reported as median (interquartile range, IQR). The Mann–Whitney U test was used to compare groups, testing the null hypothesis that the probability a randomly selected observation from one group exceeds that from the other group is 0.5 (H₀: P(X > Y) = 0.5) against the two-sided alternative (H₁: P(X > Y) ≠ 0.5). This test is distribution-free and does not assume equality of variances or specific distributional forms. Categorical variables are summarized as counts (percentages). Group comparisons were performed to assess the association between CMV reactivation status and each categorical variable: Pearson’s χ² test was used when all expected cell frequencies were ≥ 5, testing the null hypothesis of independence (H₀: no association) against the alternative of dependence (H₁: association exists). Fisher’s exact test was applied when any expected cell count was < 5, under the same null hypothesis of independence. To evaluate differences in the cumulative incidence of CMV reactivation between high- and low-risk groups (as stratified by the optimal Youden index threshold of the GBM model), Kaplan–Meier survival curves were constructed and compared using the log-rank test. This test evaluates the null hypothesis that the time-to-event (CMV reactivation) distributions are identical between the two groups (H₀: S₁(t) = S₂(t) for all t) against the two-sided alternative that at least one group has a different reactivation risk over time. All hypothesis tests were two-sided, and a p-value < 0.05 was considered statistically significant.

## Results

### Patient characteristics

A total of 221 sepsis patients admitted to the ICU were included, with 156 in the training set and 65 in the testing set (Table 1). The median age was 67.5 years, with no significant difference between groups (training: 67.5 [IQR: 59.0–75.2], test: 70.0 [IQR: 61.0–80.0]; *p* = .138). Male patients predominated (67.3%). The APACHE II score was similar between groups (training: 20.5 [IQR: 16.0–26.2], testing: 20.0 [IQR: 16.0–30.0]; *p* = .891).

The most common infection site was the lungs (59.0% and 72.3%; *p* = .174). Comorbidities such as diabetes, coronary heart disease, and chronic kidney disease showed no significant differences between groups. Laboratory markers, including CMV IgG (training: 206 AU/mL [IQR: 164–250], test: 199 AU/mL [IQR: 151–250]; *p* = .747) and CD4^+^ T lymphocyte counts (training: 304/µL [IQR: 180–458], test: 296/µL [IQR: 130–463]; *p* = .616), were also comparable.

CMV reactivation occurred in 19.0% of patients, and the 90-day mortality was 27.1%, with no significant differences between sets. The well-balanced baseline characteristics support the reliability of the model development and validation.

**Table 1:** Baseline characteristics compared between training and test sets

	Train	Test	*p*
	*N* = 156	*N* = 65	
Age, years	67.5 [59.0;75.2]	70.0 [61.0;80.0]	0.138
Gender, female(%)	51 (32.7%)	15 (23.1%)	0.207
BMI	22.7 (4.04)	23.2 (3.81)	0.395
APACHEII	20.5 [16.0;26.2]	20.0 [16.0;30.0]	0.891
Infectious sites:			0.174
Lung infection	92 (59.0%)	47 (72.3%)	
Abdominal infection	42 (26.9%)	12 (18.5%)	
Bloodstream infection	22 (14.1%)	6 (9.23%)	
DM, n(%)	38 (24.4%)	16 (24.6%)	1.000
CHD, n(%)	16 (10.3%)	9 (13.8%)	0.593
Organ transplantation, n (%)	6 (3.85%)	1 (1.54%)	0.677
CHF, n (%)	11 (7.05%)	6 (9.23%)	0.782
CKD, n(%)	19 (12.2%)	6 (9.23%)	0.691
ARDs, n(%)	13 (8.33%)	5 (7.69%)	1.000
Malignant tumor, n(%)	55 (35.3%)	22 (33.8%)	0.964
CMV IgG, AU/ml	206 [164;250]	199 [151;250]	0.747
Lymphocytes counts, x10^9/L	0.76 [0.51;1.07]	0.73 [0.43;1.00]	0.510
PLT, x10^9/L	151 [93.4;232]	148 [94.3;212]	0.689
Neu, x10^9/L	9.29 [6.66;12.6]	9.47 [6.85;11.8]	0.879
CRP, mg/L	99.1 [54.3;159]	100 [73.8;157]	0.571
ALT, IU/L	25.3 [14.3;56.4]	25.7 [12.5;41.1]	0.653
AST, IU/L	37.3 [23.1;74.8]	33.6 [25.4;80.1]	0.633
CD3^+^ lymphocyte count, /µl	513 [294;723]	440 [263;770]	0.362
CD4^+^ lymphocyte count, /µl	304 [180;458]	296 [130;463]	0.616
CD8^+^ lymphocyte count, /µl	188 [101;279]	124 [82.3;263]	0.139
APTT, s	34.1 [30.4;39.3]	33.6 [30.1;39.1]	0.586
PT, s	13.8 [12.8;15.5]	13.8 [12.5;16.0]	0.971
D-dimer, mg/L	4.70 [2.60;8.93]	4.27 [2.36;7.17]	0.357
PCT, ng/mL	0.93 [0.32;6.99]	0.99 [0.31;4.96]	0.672
Pre-alb, mg/L	115 [77.8;139]	116 [88.3;144]	0.752
Mechanical ventilation, days	10.3 (10.5)	9.77 (10.1)	0.710
Intravenous glucocorticoid, days	0.00 [0.00;7.00]	0.00 [0.00;7.00]	0.586
Vasoactive agents, days	3.00 [0.00;8.25]	4.00 [0.00;11.0]	0.740
90-day mortality, n(%)	42 (26.9%)	18 (27.7%)	1.000
CMV reactivation, n(%):	30 (19.2%)	12 (18.5%)	1.000

*Abbreviations*: BMI, body mass index; APACHE II, acute physiology and chronic health evaluation II; DM, diabetes mellitus; CAHD, coronary atherosclerotic heart disease; CHF, chronic heart failure; CKD, chronic kidney disease; AIDs, autoimmune diseases; CMV, cytomegalovirus; ALT, alanine transaminase; AST, aspartate transaminase; APTT, activated partial thromboplastin time; PT, prothrombin time; PCT, procalcitonin

### Feature selection

Univariate logistic regression was used to select significant predictors for CMV reactivation (Table 2). Variables with p-values ≤ 0.05 were incorporated into the machine learning models. The variables selected included APACHE II score, CMV IgG level, history of organ transplantation, chronic kidney disease (CKD), CD4^+^ lymphocyte count, and the number of days of intravenous corticosteroid use within 28 days.

**Table 2:** Univariable logistic regression of CMV reactivation risk factors in training dataset

CMV reactivation	OR (univariable)
Age, years	0.99 (0.96–1.01, *p* = .304)
Gender, male(%)	1.04 (0.44–2.41, *p* = .934)
BMI	0.98 (0.89–1.09, *p* = .722)
APACHEII	1.05 (1.00-1.09, *p* = .050)
Infectious site	0.88 (0.34–2.32, *p* = .800)
	1.65 (0.56–4.85, *p* = .359)
DM, n(%)	0.73 (0.28–1.96, *p* = .537)
CHD, n(%)	0.57 (0.12–2.66, *p* = .476)
Organ transplantation, n(%)	9.54 (1.66–54.85, *p* = .011)
CHF, n(%)	2.62 (0.71–9.59, *p* = .147)
CKD, n(%)	2.89 (1.03–8.13, *p* = .044)
ARDs, n(%)	2.00 (0.57-7.00, *p* = .278)
Malignant tumor, n(%)	0.61 (0.25–1.48, *p* = .276)
CMV IgG, AU/ml	1.02 (1.01–1.03, *p* = .003)
Lymphocytes counts, x10^9/L	0.55 (0.22–1.36, *p* = .195)
PLT, x10^9/L	1.00 (0.99-1.00, *p* = .496)
Neu, x10^9/L	1.01 (0.93–1.09, *p* = .855)
CRP, mg/L	0.99 (0.99-1.00, *p* = .090)
ALT, IU/L	1.00 (0.99-1.00, *p* = .250)
AST, IU/L	1.00 (1.00–1.00, *p* = .654)
CD3^+^ lymphocyte count, /µl	1.00 (1.00–1.00, *p* = .180)
CD4^+^ lymphocyte count, /µl	1.00 (0.99-1.00, *p* = .018)
CD8^+^ lymphocyte count, /µl	1.00 (1.00–1.00, *p* = .914)
APTT, s	1.00 (0.95–1.05, *p* = .955)
PT, s	1.10 (0.97–1.24, *p* = .126)
D-dimer, mg/L	1.00 (0.94–1.07, *p* = .912)
PCT, ng/mL	1.00 (0.98–1.02, *p* = .878)
Pre-alb, mg/L	1.01 (1.00-1.02, *p* = .057)
Mechanical ventilation, days	1.02 (0.98–1.05, *p* = .394)
Intravenous glucocorticoid, days	1.05 (1.00-1.10, *p* = .049)
Vasoactive agents, days	1.04 (0.99–1.09, *p* = .130)

*Abbreviations*: BMI, body mass index; APACHE II, acute physiology and chronic health evaluation II; DM, diabetes mellitus; CAHD, coronary atherosclerotic heart disease; CHF, chronic heart failure; CKD, chronic kidney disease; AIDs, autoimmune diseases; CMV, cytomegalovirus; ALT, alanine transaminase; AST, aspartate transaminase; APTT, activated partial thromboplastin time; PT, prothrombin time; PCT, procalcitonin

### Model performance

**Fig. 2:**
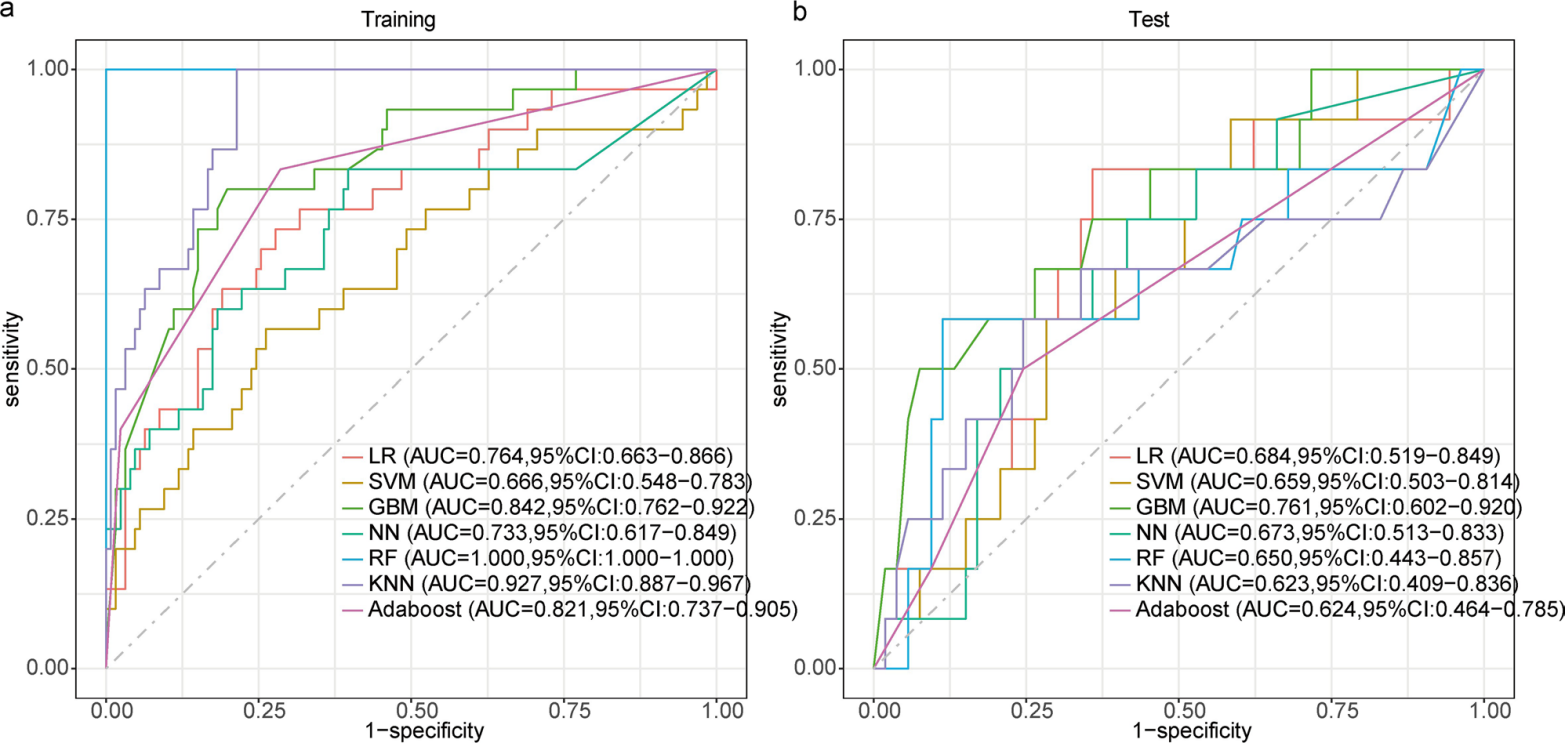
ROC curve of seven models model for CMV reactivation in the (**a**) training and (**b**) test datasets. *Abbreviations*: GBM, gradient boosting machine. *Abbreviations*: ROC, Receiver operating characteristic; AUC, area under the curve; LR, logistic regression; SVM, support vector machine; GBM, gradient boosting machine; NN, neural network; RF, random forest; KNN, k-nearest neighbors; AdaBoost, adaptive boosting

Six machine learning algorithms were trained: LG, SVM, GBM, NN, RF, and KNN, and AdaBoost. The 10-fold cross-validated performance (repeated five times) on the training set is reported in Supplementary Table 4 as mean ± SD. Model performance is summarized as point estimates (95% CIs) in Table 3, with the GBM model achieving the highest AUC in the test set (Fig. 2). The AUC value for the GBM model was 0.76, with its confusion matrices shown in Supplementary Fig. 1. The next best-performing models were LG and NN.

**Table 3:** Evaluation metrics of seven models

Model	Threshold	Accuracy	Sensitivity	Specificity	Precision	F1 Score	AUC
Traing							
LR	0.2	0.724 (0.654–0.795)	0.733 (0.576–0.900)	0.722 (0.637–0.800)	0.386 (0.264–0.519)	0.506 (0.372–0.636)	0.764 (0.663–0.866)
SVM	0.182	0.705 (0.635–0.776)	0.567 (0.391–0.750)	0.738 (0.658–0.814)	0.340 (0.220–0.475)	0.425 (0.286–0.550)	0.666 (0.548–0.783)
GBM	0.26	0.801 (0.737–0.865)	0.800 (0.650–0.933)	0.802 (0.732–0.871)	0.490 (0.357–0.630)	0.608 (0.471–0.723)	0.842 (0.762–0.922)
NN	0.155	0.647 (0.571–0.718)	0.833 (0.679–0.963)	0.603 (0.512–0.680)	0.333 (0.236–0.443)	0.476 (0.362–0.593)	0.733 (0.617–0.849)
RF	0.5	1.000 (-)	1.000 (-)	1.000 (-)	1.000 (-)	1.000 (-)	1.000 (1.000–1.000)
KNN	0.221	0.827 (0.769–0.885)	1.000 (-)	0.786 (0.711–0.857)	0.526 (0.393–0.660)	0.690 (0.564–0.795)	0.927 (0.887–0.967)
AdaBoost	0.165	0.737 (0.667–0.808)	0.833 (0.700–0.963)	0.714 (0.636–0.794)	0.410 (0.290–0.537)	0.549 (0.419–0.660)	0.821 (0.737–0.905)
Test							
LR	0.173	0.677 (0.554–0.785)	0.833 (0.583–1.000)	0.642 (0.509–0.765)	0.345 (0.171–0.517)	0.488 (0.273–0.666)	0.684 (0.519–0.849)
SVM	0.177	0.508 (0.385–0.631)	0.917 (0.715–1.000)	0.415 (0.286–0.538)	0.262 (0.122–0.395)	0.407 (0.213–0.557)	0.659 (0.503–0.814)
GBM	0.427	0.846 (0.754–0.923)	0.500 (0.200–0.800)	0.925 (0.847–0.982)	0.600 (0.250–0.900)	0.545 (0.250–0.769)	0.761 (0.602–0.920)
NN	0.181	0.723 (0.600–0.831)	0.583 (0.267–0.889)	0.755 (0.623–0.864)	0.350 (0.143–0.571)	0.438 (0.194–0.632)	0.673 (0.513–0.833)
RF	0.445	0.831 (0.738–0.908)	0.583 (0.267–0.889)	0.887 (0.800–0.963)	0.538 (0.250–0.812)	0.560 (0.267–0.769)	0.650 (0.443–0.857)
KNN	0.316	0.723 (0.615–0.831)	0.583 (0.267–0.889)	0.755 (0.633–0.864)	0.350 (0.136–0.571)	0.438 (0.191–0.643)	0.623 (0.409–0.836)
AdaBoost	0.165	0.708 (0.600–0.815)	0.500 (0.200–0.818)	0.755 (0.642–0.868)	0.316 (0.106–0.545)	0.387 (0.154–0.606)	0.624 (0.464–0.785)

*Abbreviations*: AUC, area under the curve; LR, logistic regression; SVM, support vector machine; GBM, gradient boosting machine; NN, neural network; RF, random forest; KNN, k-nearest neighbors; AdaBoost, adaptive boosting

**Fig. 3:**
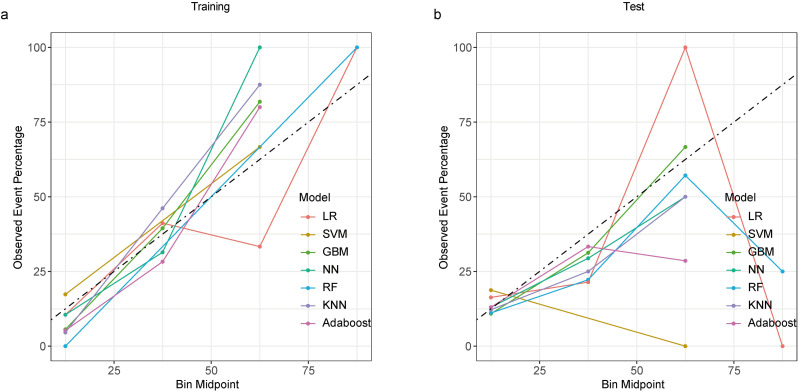
Calibration performance of seven models in the (**a**) training and (**b**) test datasets. *Abbreviations*: LR, logistic regression; SVM, support vector machine; GBM, gradient boosting machine; NN, neural network; RF, random forest; KNN, k-nearest neighbors; AdaBoost, adaptive boosting

**Fig. 4:**
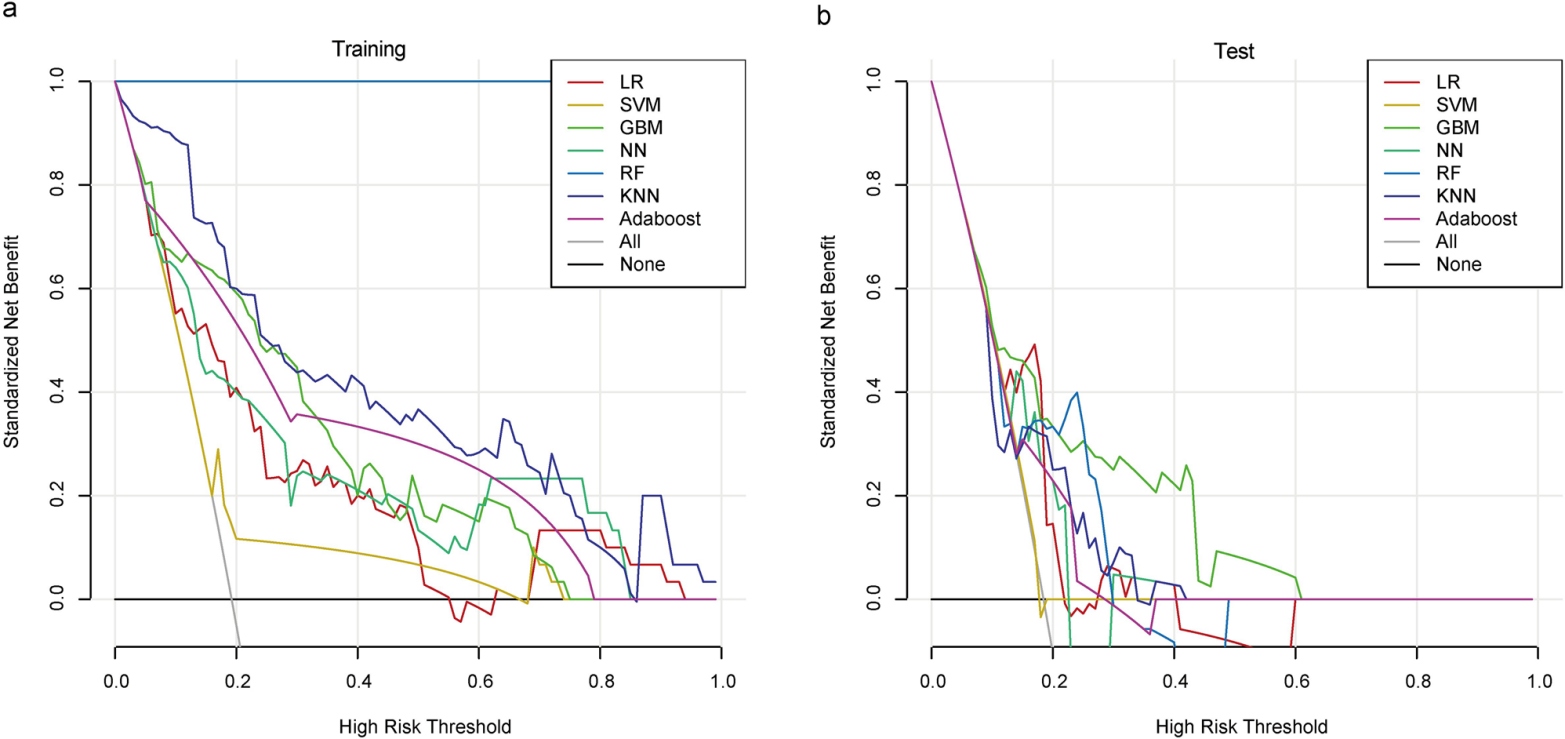
DCA of seven models for both (**a**)training and (**b**)test datasets. *Abbreviations*: DCA, decision curve analysis, LR, logistic regression; SVM, support vector machine; GBM, gradient boosting machine; NN, neural network; RF, random forest; KNN, k-nearest neighbors; AdaBoost, adaptive boosting

The calibration plot for the GBM model showed a slope close to the ideal 1:1 line, indicating good calibration between predicted and observed risk (Fig. 3). Additionally, DCA was performed, where the grey line represents the hypothetical scenario of all patients receiving intervention and the black horizontal line represents no patients receiving intervention. Given the heterogeneity of the study population, intervention strategies based on any of the seven machine learning models outperformed the standard strategies of treating all or no patients. The net benefit of the GBM model in the test set exceeded that of the other models at threshold probabilities ranging from 10% to 60% (Fig. 4). As expected in predictive modeling, the net benefit observed in DCA was consistently lower in the test set than in the training set across all seven models, reflecting the inherent optimism in training performance. Despite this general decline, the GBM model maintained a relatively higher net benefit compared to other approaches throughout the clinically relevant threshold range (10%–60%), underscoring its robustness even in a limited sample. Given the size of our cohort (*n* = 221) and single-center design, the observed DCA attenuation may also stem from statistical variability and limited event counts (*n* = 42 CMV reactivations). Future validation in larger, multicenter datasets will be essential to confirm the generalizability and clinical utility of the GBM-based decision strategy.

**Fig. 5:**
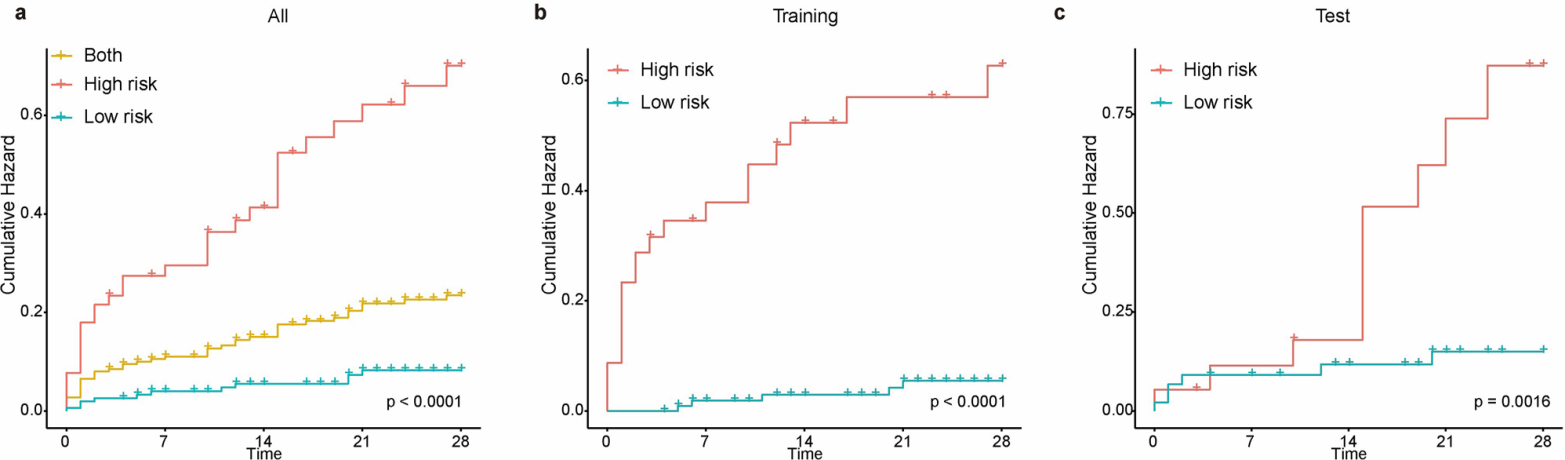
Kaplan-Meier curves of CMV reactivation incidence within 28 days for patients with low and high machine learning risk in the (**a**) all, (**b**) training, and (**c**) test cohorts

The GBM model was then applied to predict and stratify CMV reactivation risk in the test set. Patients were classified into high-risk and low-risk groups based on the optimal cutoff value (0.260482) determined by the maximum Youden index. Kaplan-Meier analysis revealed a significantly higher reactivation rate within 28 days in the high-risk group compared to the low-risk group, highlighting that patients with higher predicted scores were more likely to experience CMV reactivation (log-rank test: *p* < .05) (Fig. 5). Notably, the Kaplan–Meier curves in the training and test sets show slight differences in the timing and steepness of divergence between risk groups. These differences likely stem from the small test set size, which increases variability in time-to-event estimates—even with balanced baseline characteristics (Table 1). The GBM-derived Youden cutoff, optimized on the training data, may not perfectly align with absolute risk timing in a smaller cohort with low event rates. Nonetheless, significant log-rank separation in both sets confirms the model’s ability to reliably stratify patients into clinically meaningful risk groups.

### Visualization of feature importance

**Fig. 6:**
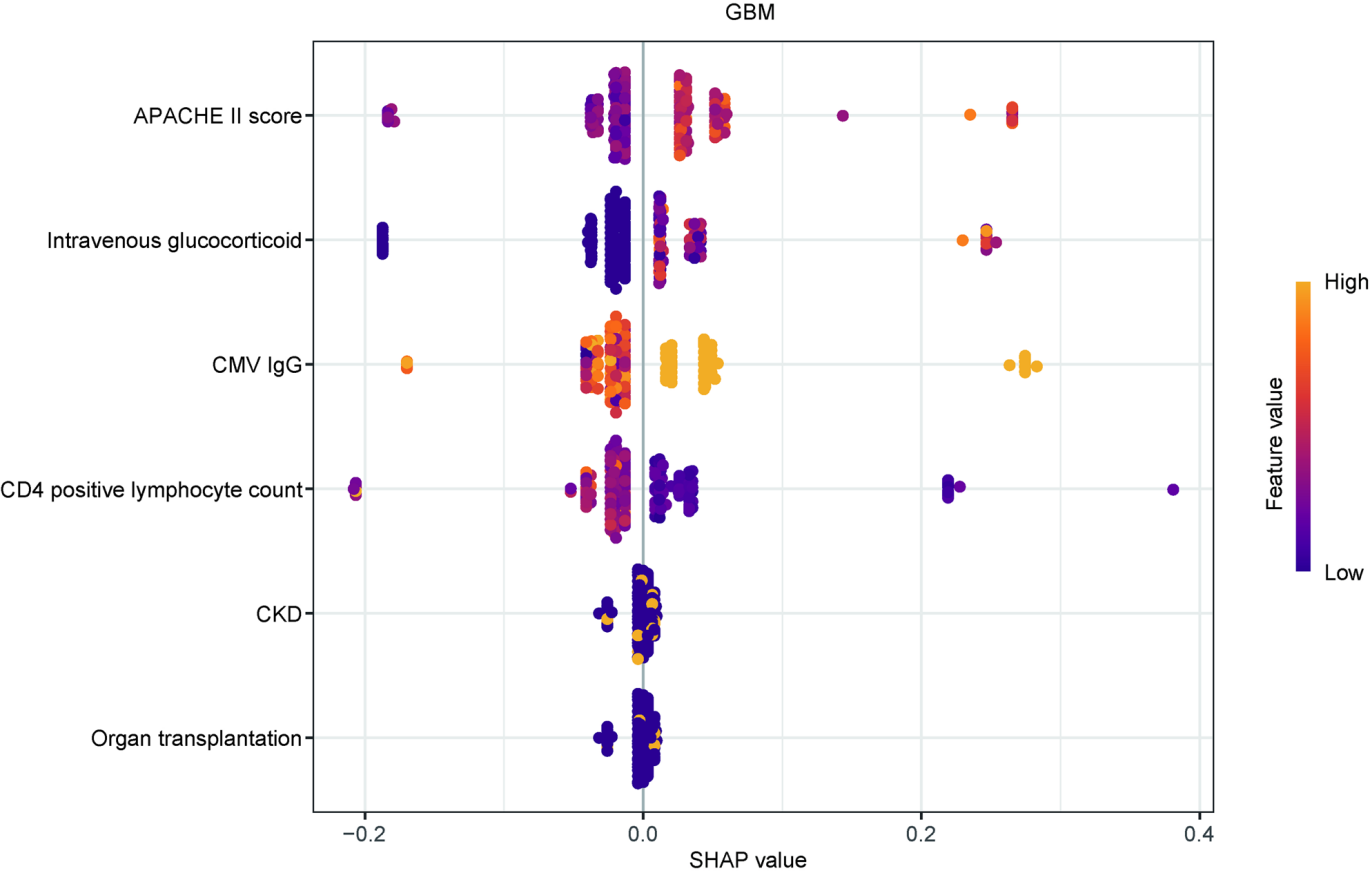
Importance ranking of selected predictors with stability and interpretation using the optimal model. A higher SHAP value for a feature indicates a higher risk of CMV reactivation. The yellow portion of the feature value represents higher original values

To gain a deeper understanding of the selected variables, SHAP was employed to visualize how these factors influenced the predictions in the GBM model. Figure 6 presents the SHAP summary plot, with the feature ranking (y-axis) indicating the relative importance of each variable in the model (mean absolute SHAP in Supplementary Fig. 2 and variable-SHAP relations in Supplementary Fig. 3). The SHAP values (x-axis) represent the contribution of each feature to the prediction, with red dots corresponding to high-risk values and blue dots to low-risk values. Key predictors, including higher APACHE II scores, longer durations of intravenous corticosteroid use, elevated CMV IgG titers, lower CD4^+^ lymphocyte counts, and histories of chronic kidney disease or organ transplantation, were associated with increased predicted probabilities of CMV reactivation. Notably, the ranking of feature importance derived from SHAP values differs somewhat from the variable ordering based on univariate logistic regression odds ratios (Table 2). This discrepancy may arise because univariate analysis evaluates each predictor in isolation, without accounting for interactions or correlations among variables, whereas SHAP values reflect each feature’s contribution within the full GBM model, which offers both a global perspective (via the mean absolute SHAP value) and local, patient-specific explanations that account for the influence of other covariates in each individual’s context. As a result, SHAP provides a more context-aware assessment of each variable’s role in CMV reactivation risk—offering insights that may reasonably differ from those obtained through simpler univariate analyses.

### Interpretation of personalized predictions

**Fig. 7:**
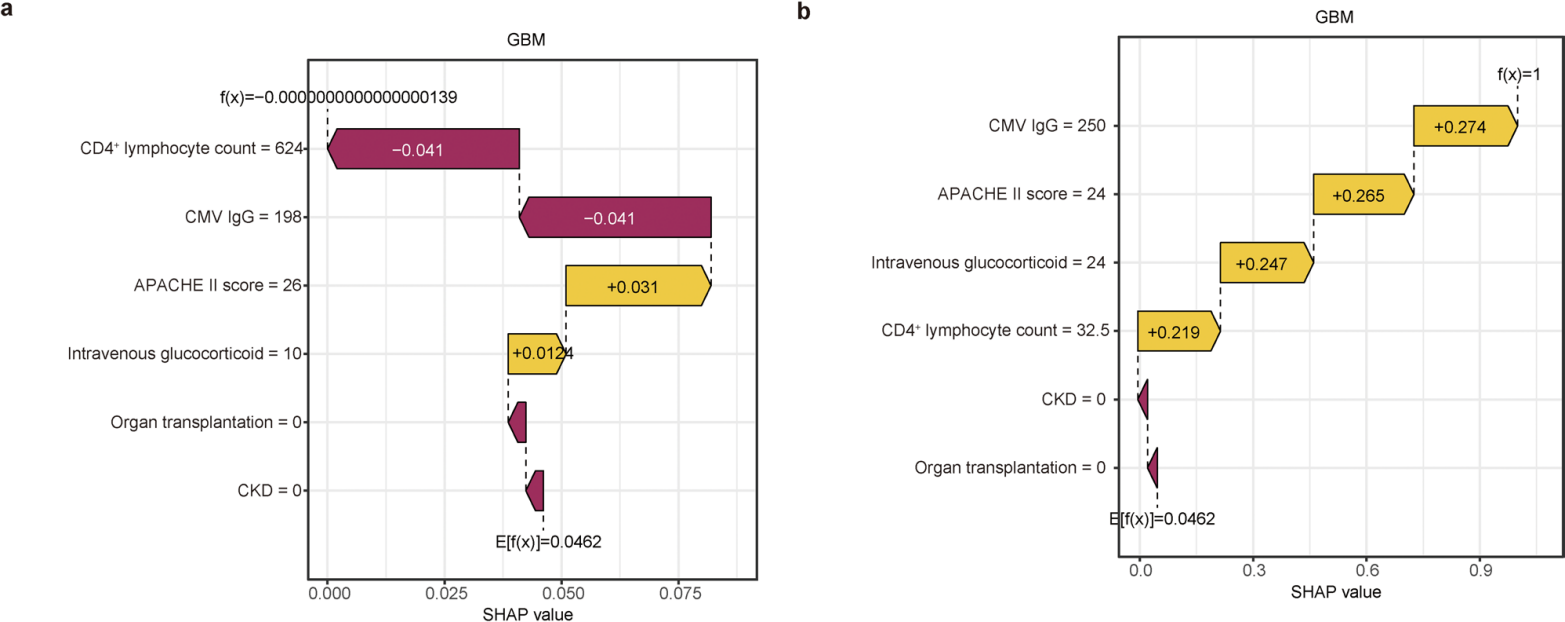
Waterfall plots for interpretation of model prediction results with a non-reactivation patient (**a**) and a reactivation patient (**b**). *Abbreviations*: APACHE II, acute physiology and chronic health evaluation II score; CKD, chronic kidney disease

Figure 7 displays individual SHAP waterfall plots for (a) non-reactivated patients and (b) reactivated patients. These plots illustrate how each feature contributed to the predicted probability for each individual. The numbers represent the predicted probability values (*f(x)*), while the base value is the average prediction without model inputs. Here, *f(x)* reflects the log odds ratio for each observation. Features highlighted in yellow indicate increased risk, whereas those in red signify decreased risk. The length of the arrows represents the magnitude of each feature’s impact, with longer arrows signifying greater influence on the prediction.

## Discussion

In this retrospective study, we developed a machine learning-based model to predict CMV reactivation in ICU patients with sepsis. Among the models evaluated, the GBM model demonstrated the best performance. High-risk group defined by the model output were more likely to experience CMV reactivation in all, training, and test cohorts. These findings highlighting its potential as a reliable tool for risk stratification and treatment optimization. Furthermore, the incorporation of SHAP values provided valuable insights into the contributions of each clinical feature to the predictive outcomes. The visual interpretation of feature importance allows clinicians to easily understand the features included in the GBM model.

In patients with sepsis, the pooled rate of CMV reactivation is approximately 30% [17]. In our dataset, CMV reactivation occurred in 21% of patients within 28 days of ICU admission (Supplementary Table 5), with more than half of these cases occurring within the first 7 days (Supplementary Fig. 4). These findings align with those of a prospective study on CMV reactivation conducted across various ICU types in two centers, which reported CMV viremia incidence ranging from 15% to 65% within 10 days [6]. Additionally, CMV reactivation has been associated with worse clinical outcomes, including higher mortality rates, prolonged ICU and hospital stays, extended duration of mechanical ventilation, and increased incidence of secondary infections [2–6, 18–21]. As shown in Supplementary Table 5, patients with CMV reactivation had a higher number of days on vasoactive agents and an increased 90-day mortality rate.

Our findings demonstrate both consistencies and differences when compared to existing studies on risk factors for CMV reactivation. In this study, the APACHE II score emerged as the most significant predictor of CMV reactivation risk. This aligns with previous research in sepsis populations, which has demonstrated a positive correlation between disease severity and CMV reactivation rates [2, 3], a similar trend also noted in COVID-19 populations [22]. However, this association has not been observed in larger or different cohorts [6, 23]. Notably, studies that adjusted for baseline disease severity still reported a correlation between CMV reactivation and an increased risk of worse clinical outcomes [21].

In sepsis patients, immune suppression involves a complex interplay of mechanisms, including immune cell exhaustion and apoptosis, reprogramming of antigen-presenting cells, and disruptions in epigenetic regulatory pathways [24]. These profound alterations in immune function may predispose individuals to CMV reactivation from latency, potentially explaining why this phenomenon is more prominent in sepsis compared to other conditions with differing immune profiles.

Corticosteroids are commonly used in the management of sepsis and other immune-related conditions. Many previous studies have excluded patients receiving systemic corticosteroid therapy [23]. However, the latest Surviving Sepsis Campaign (SSC) guidelines recommend intravenous corticosteroids for adults with septic shock who require ongoing vasopressor support. As a result, our study included such patients to better reflect real-world clinical practices. In our study, the number of days of intravenous corticosteroid use within the first 28 days of ICU admission was significantly associated with CMV reactivation. This association may be attributed not only to the immunosuppressive effects of corticosteroids but also to their potential role in promoting CMV gene expression [25].

Immune dysregulation is a critical factor driving CMV reactivation in sepsis patients. Studies have shown that cell-mediated immunity (CMI) plays a critical role in controlling CMV replication. In hematopoietic stem cell transplantation (HSCT) patients, those with higher CMV IgG titers are at increased risk of CMV reactivation [26–28]. Elevated anti-CMV IgG levels may reflect more frequent subclinical CMV reactivations and inadequate control of viral replication [29]. Additionally, patients with higher CMV IgG levels may harbor more active viral strains, which increases their susceptibility to reactivation following HSCT [30].

Many studies have explored the role of CD8^+^ T cells in CMV infection and reactivation [17, 31, 32]. However, investigations into CD4^+^ T cells are less common and primarily focus on organ transplant populations. CD4^+^ T cells are well-known for their auxiliary role in supporting CD8^+^ T cell and B cell responses. Our findings suggest that a low absolute count of CD4^+^ lymphocytes is also a risk factor for CMV reactivation. During CMV viremia, the levels of CMV-specific CD4^+^ T cells significantly decrease but recover after the resolution of viremia. Notably, CMV-specific T cell levels begin to decline even before viral loads become detectable, whereas CD8^+^ T cell counts remain stable throughout [33]. In stem cell transplant (SCT) populations, CD4^+^ T cells and natural killer (NK) cells—rather than CD8^+^ T cells—have been associated with CMV reactivation prevention [34]. CD4^+^ T cells play a crucial role in the induction, activation, and maintenance of CD8^+^ T cell function [31]. Some studies have also identified cytotoxic CD4^+^ T lymphocytes, suggesting their potential dual roles in viral infections [35, 36]. Furthermore, CD4^+^ T cells control viral replication through the production of IFN-γ and the immunosuppressive cytokine IL-10 [24]. Finally, M25-specific CD4^+^ tissue-resident memory cells play a crucial role in latency and preventing CMV reinfection [37].

Risk factors for CMV reactivation share commonalities across patient populations, particularly the roles of immunosuppression and disease severity. However, their impact can vary significantly due to differences in underlying conditions, treatment regimens, and immune response mechanisms across patient groups. These variations underscore the importance of considering the specific clinical and treatment context when evaluating risk. Currently, there is no conclusive evidence supporting the routine use of prophylactic anti-CMV drugs, such as ganciclovir, valganciclovir or novel prophylactic agents, to prevent CMV reactivation in ICU patients. While preemptive therapy and prophylaxis with antiviral agents have been a general agreement in HSCT populations [38, 39], careful consideration of potential side effects—such as hematologic toxicity and renal impairment—is necessary in ICU population. What’s more, compared to the general transplant population, critically ill patients face a lower percentage of CMV recurrence [40], which makes the application of prophylactic antiviral drugs to the entire ICU sepsis population, as is commonly done in transplant recipients, offer less benefit [41]. Therefore, a predictive model to identify patients at high risk for CMV reactivation is essential for tailoring antiviral strategies, guiding prophylactic interventions, and avoiding both overtreatment and delays in care, ultimately ensuring the best outcomes for ICU patients.

This study has several limitations. First, as a retrospective, single-center study based on existing medical records, there are inherent challenges related to data quality control, which may introduce selection bias and information bias. Since the model was trained and validated using data from a single center, its generalizability to other settings has not been tested, limiting its applicability across different populations. Additionally, the study did not account for the use of antiviral medications, such as prior routine administration or ICU treatments, which could impact CMV reactivation rates and patient outcomes. Future research should explore the influence of antiviral therapy on outcomes in patients with CMV reactivation to refine risk stratification and intervention strategies. Second, this study primarily relied on structured data. Future work could incorporate unstructured data, such as clinical notes, additional risk indicators, imaging data, environmental factors, and lifestyle information, to further enhance predictive performance. Collecting routinely available clinical data could also improve the model’s practicality and ease of implementation. The incorporation of deep learning techniques could enable more sophisticated data analysis and model refinement, thereby improving the accuracy of CMV reactivation predictions. Third, our study faced challenges related to class imbalance, limited sample size, and a relatively high number of candidate features. With only 42 CMV reactivation events among 221 patients, the positive class was markedly underrepresented, which may bias model training toward the majority class. To address this imbalance, we applied the Synthetic Minority Over-sampling Technique (SMOTE) during preprocessing. However, training on the SMOTE-augmented dataset did not lead to improved model performance compared to training on the original (non-SMOTE) dataset (Supplementary Table 6). Moreover, the ratio of events per variable (EPV) was low—approximately 7:1 given the six selected predictors—falling below the commonly recommended threshold of 10:1 for stable logistic modeling and potentially affecting the reliability of feature selection and model calibration. Moreover, the ratio of events per variable (EPV) was low—approximately 7:1 given the six selected predictors—falling below the commonly recommended threshold of 10:1 for stable logistic modeling and potentially affecting the reliability of feature selection and model calibration. Nonetheless, we hope that our transparent variable selection process, combined with the use of an interpretable machine learning framework (SHAP) and consistent test-set performance, provides a reasonable balance given the current sample size.

Overall, this study provides a novel approach for predicting CMV reactivation in sepsis patients and highlights several avenues for future improvements. With continued model optimization and refinement, this approach has the potential to become an essential tool in clinical practice for the prevention and management of CMV reactivation.

## Conclusions

In this study, we developed a machine learning model to predict CMV reactivation in critically ill sepsis patients, achieving good performance with GBM model. This model offers a promising tool for risk stratification, potentially guiding individualized interventions of CMV reactivation. Future research should aim to validate the model in larger, multicenter cohorts and incorporate additional data sources and features to improve its generalizability and clinical applicability.

## Supplementary Information

Below is the link to the electronic supplementary material.

Supplementary Material 1

Supplementary Material 2

Supplementary Material 3

Supplementary Material 4

Supplementary Material 5

Supplementary Material 6

Supplementary Material 7

Supplementary Material 8

Supplementary Material 9

Supplementary Material 10

Supplementary Material 11

## Data Availability

The datasets analyzed during the current study are not publicly available due to patient privacy but are available from the corresponding author on reasonable request.

## Abbreviations

CMV: Cytomegalovirus
ICU: Intensive Care Unit
APACHE II: Acute Physiology and Chronic Health Evaluation II
GBM: Gradient Boosting Machine
SHAP: SHapley Additive exPlanations
LR: Logistic Regression
NN: Neural Network
SVM: Support Vector Machine
RF: Random Forest
AdaBoost: Adaptive Boosting
KNN: K-Nearest Neighbors
ROC: Receiver Operating Characteristic
AUC: Area Under the Curve
DCA: Decision Curve Analysis
Youden Index: Youden Index
IFN-γ: Interferon-gamma
HSCT: Hematopoietic Stem Cell Transplantation
CMI: Cell-Mediated Immunity
HIV: Human Immunodeficiency Virus
CAD: Coronary Artery Disease
CKD: Chronic Kidney Disease
APTT: Activated Partial Thromboplastin Time
PT: Prothrombin Time
PCT: Procalcitonin
AST: Aspartate Aminotransferase
ALT: Alanine Aminotransferase
NK: Natural Killer
SSC: Surviving Sepsis Campaign
SMOTE: Synthetic Minority Over-sampling Technique
