# Supplementary material for: Risk stratification for CMV reactivation in sepsis patients: development of an interpretable machine learning model
Source: BMC Infect Dis. 2025 Dec 22;25:1729. doi: 10.1186/s12879-025-12154-0 (PMC12723881; doi:10.1186/s12879-025-12154-0)
Supplement: Supplementary file 1 — Supplementary Material 1 [file 12879_2025_12154_MOESM1_ESM.docx]

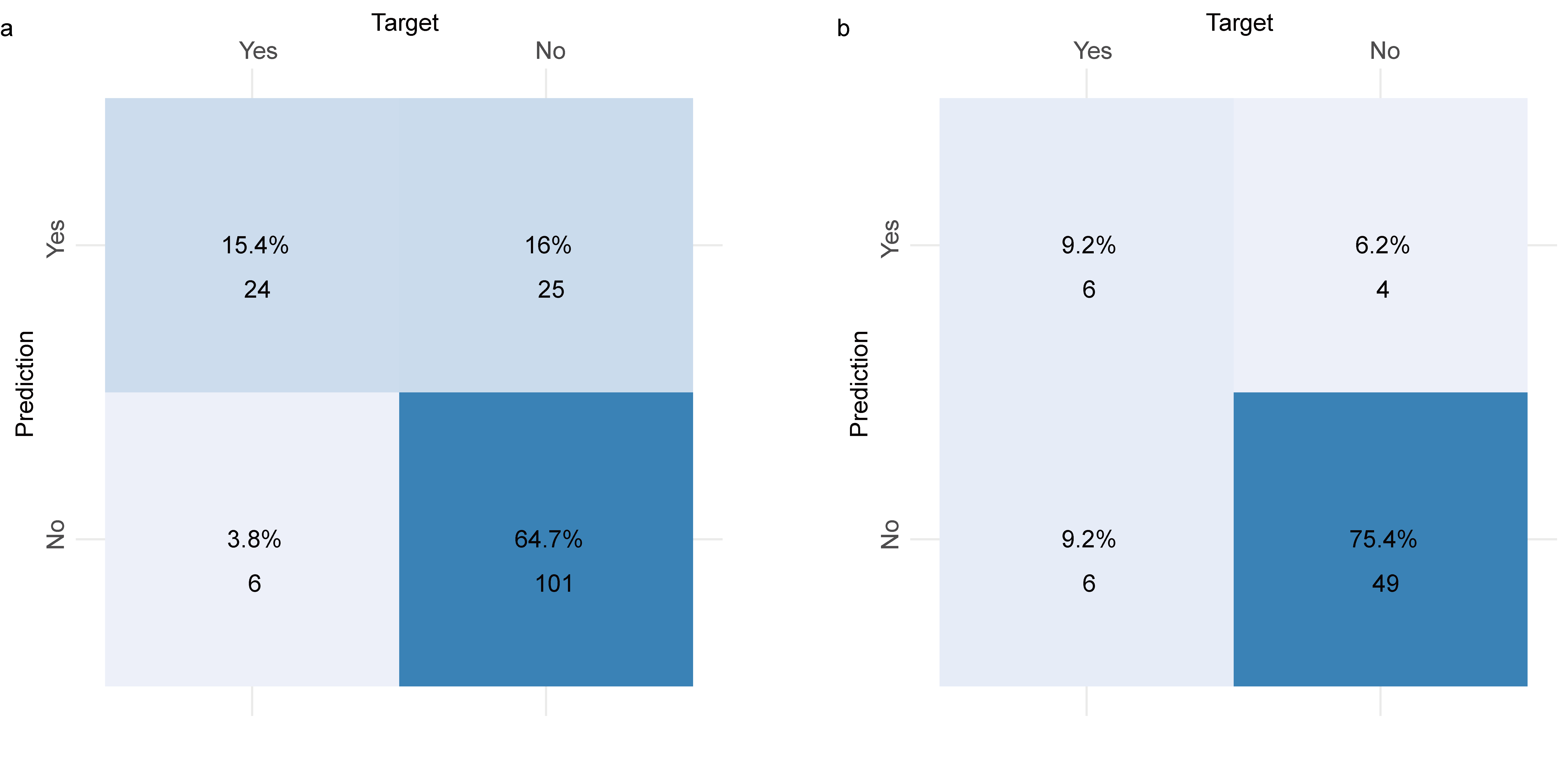


**Supplementary Figure 1.** Confusion matrices for the training set (a) and test set (b), showing the distribution of true positives, true negatives, false positives, and false negatives.


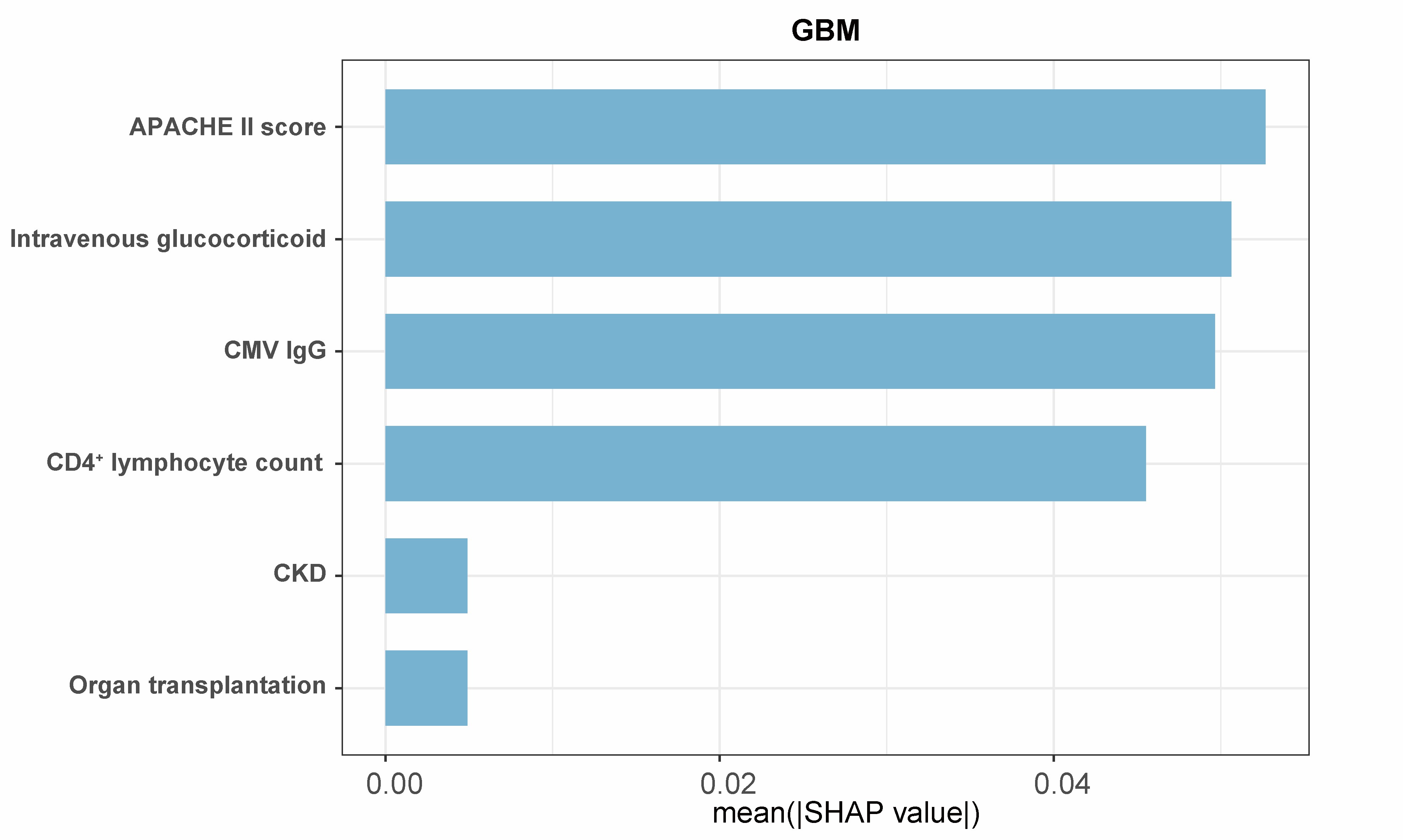


**Supplementary Figure 2.** Importance ranking of the selected variables based on the mean absolute SHAP value (|SHAP value|).


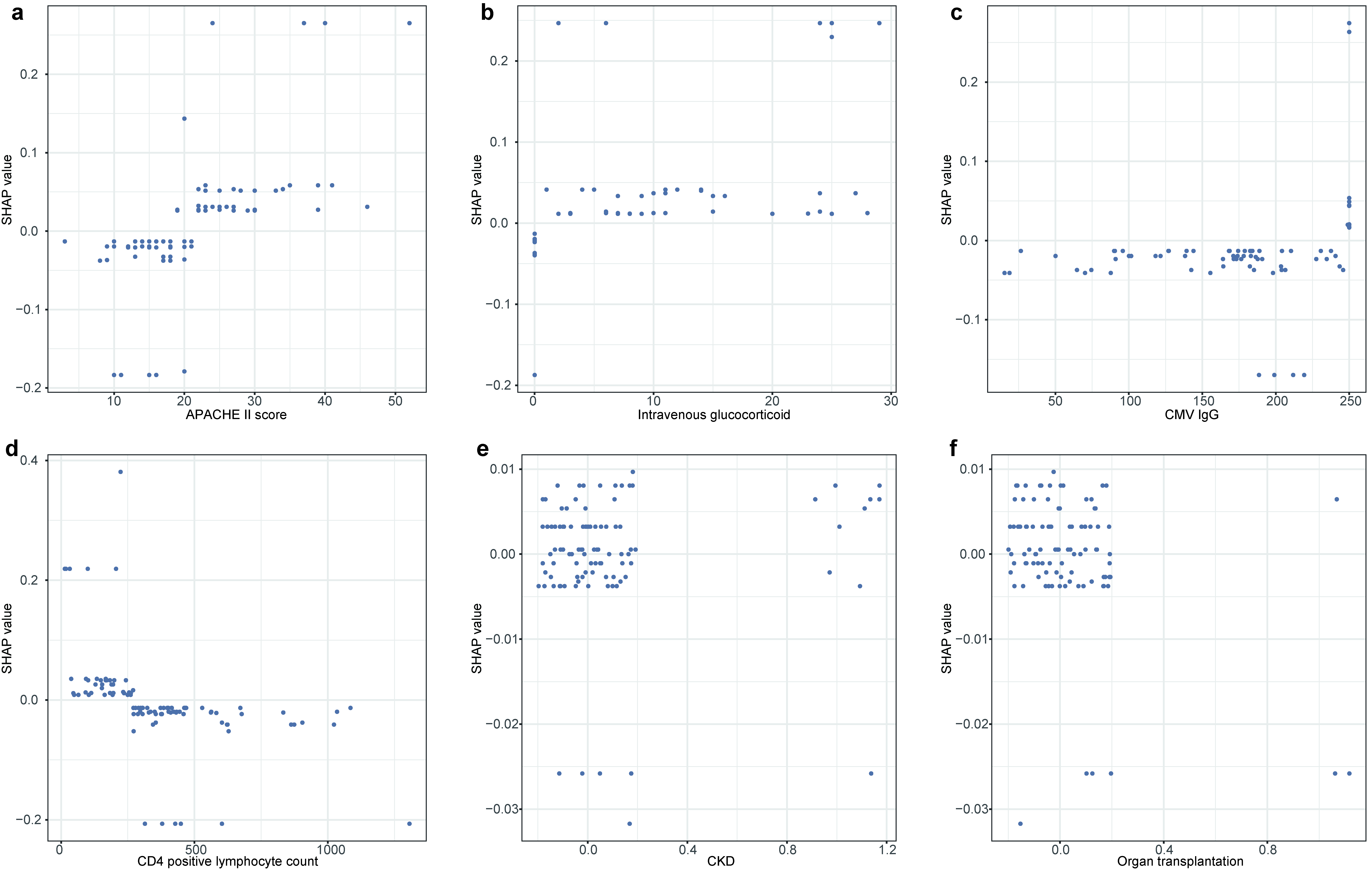


**Supplementary Figure 3.** Dependence plots showing how a single feature affects the output of the GBM prediction model.


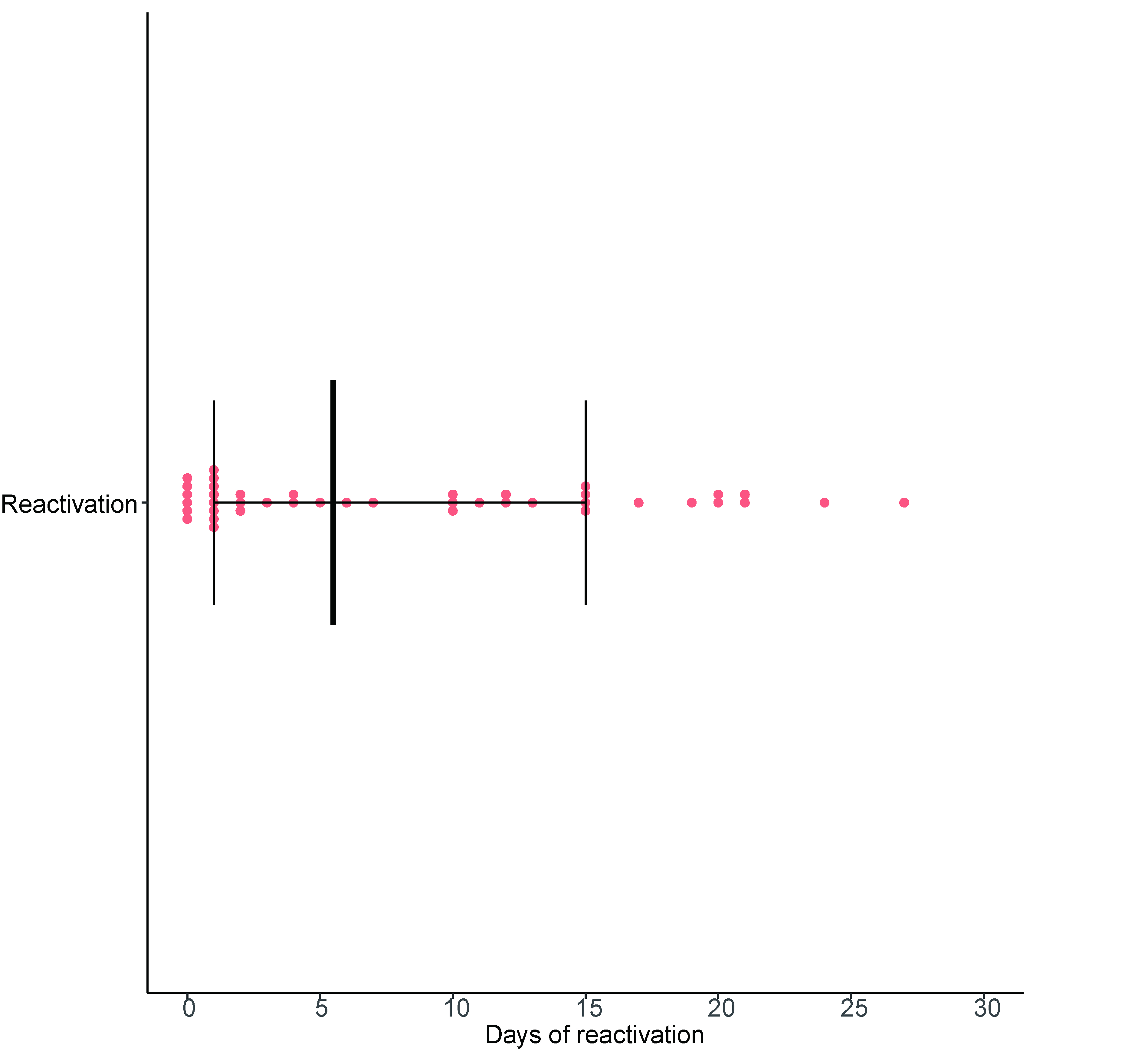


**Supplementary Figure 4.** Swarm plot showing the distribution of reactivation events over days of reactivation. The vertical black line indicates the median day of reactivation, with error bars representing the interquartile range (IQR, upper and lower quartiles).

**Supplementary Table 1.** Sequential Organ Failure Assessment Score

| **System** | **Score** | | | | |
| --- | --- | --- | --- | --- | --- |
|  | **0** | **1** | **2** | **3** | **4** |
| PaO_2_/FIO_2_, mm Hg   (kPa) | ≥400 (53.3) | <400 (53.3) | <300 (40) | <200 (26.7) with respiratory support | <100 (13.3) with respiratory support |
| Platelets, ×10^3^/µL | ≥150 | <150 | <100 | <50 | <20 |
| Bilirubin, mg/dL   (µmol/L) | <1.2 (20) | 1.2–1.9 (20–32) | 2.0–5.9 (33–101) | 6.0–11.9 (102–204) | >12.0 (204) |
| Cardiovascular  (µg/kg/min) | MAP ≥70 mm Hg | MAP <70 mm Hg | Dopamine <5 or dobutamine (any dose) | Dopamine 5.1–15 or epinephrine ≤0.1 or norepinephrine ≤0.1 | Dopamine >15 or epinephrine >0.1 or norepinephrine >0.1 |
| Glasgow Coma Scale score | 15 | 13–14 | 10–12 | 6–9 | <6 |
| Creatinine, mg/dL   (µmol/L) | <1.2 (110) | 1.2–1.9 (110–170) | 2.0–3.4 (171–299) | 3.5–4.9 (300–440) | >5.0 (440) |
| Urine output, mL/d |  |  |  | <500 | <200 |

*Abbreviations*: FIO_2_, fraction of inspired oxygen; MAP, mean arterial pressure; PaO_2_, partial pressure of oxygen.

**Supplementary Table 2.** Key hyperparameters used in models

| **Model** | **Hyperparameter** | **Value(s)** |
| --- | --- | --- |
| LR | Not applied | Not applied |
| SVM | sigma | 0.001 |
|  | C | 0.09 |
| GBM | n.trees | 100 |
|  | interaction.depth | 5 |
|  | shrinkage | 0.1 |
|  | n.minobsinnode | 30 |
| NN | size | 6 |
|  | decay | 0.6 |
| RF | mtry | 11 |
|  | numRandomCuts | 3 |
| KNN | kmax | 12 |
|  | distance | 1 |
|  | kernel | optimal |
| AdaBoost | mfinal | 2 |
|  | maxdepth | 2 |
|  | coeflearn | Zhu |

*Abbreviations:* LR, logistic regression; SVM, support vector machine; GBM, gradient boosting machine; NN, neural network; RF, random forest; KNN, k-nearest neighbors; AdaBoost, adaptive boosting.

**Supplementary Table 3.** Mainly R packages with versions used.

| **Package** | **Version** |
| --- | --- |
| caret | 7.0-1 |
| e1071 | 1.7-16 |
| gbm | 2.2.2 |
| nnet | 7.3-19 |
| extraTrees | 1.0.5 |
| kknn | 1.3.1 |
| adabag | 5.0 |
| stats | 4.3.3 |
| pROC | 1.18.5 |
| ROCR | 1.0-11 |
| boot | 1.3-31 |
| Metrics | 0.1.4 |
| DALEX | 2.4.3 |
| kernelshap | 0.5.0 |
| shapviz | 0.9.6 |
| rmda | 1.6 |
| dcurves | 0.5.0 |
| ResourceSelection | 0.3-6 |
| DynNom | 5.1 |
| ROSE | 0.0-4 |
| DMwR | 0.4.1 |
| compareGroups | 4.9.1 |

**Supplementary Table 4.** 10-Fold Cross-Validated Performance Metrics with Mean ± Standard Deviation for Training Set

| **Model** | **Accuracy** | **Sensitivity** | **Specificity** | **Precision** | **F1 Score** | **AUC** |
| --- | --- | --- | --- | --- | --- | --- |
| LR | 0.801±0.060 | 0.140±0.166 | 0.959±0.068 | 0.535±0.454 | 0.447±0.083 | 0.727±0.156 |
| SVM | 0.802±0.018 | 0.027±0.091 | 0.987±0.030 | 0.250±0.267 | 0.400±0.000 | 0.619±0.202 |
| GBM | 0.818±0.062 | 0.193±0.203 | 0.967±0.051 | 0.624±0.428 | 0.501±0.121 | 0.759±0.157 |
| NN | 0.799±0.055 | 0.140±0.214 | 0.957±0.063 | 0.447±0.387 | 0.479±0.136 | 0.615±0.205 |
| RF | 0.769±0.086 | 0.280±0.246 | 0.886±0.094 | 0.407±0.332 | 0.447±0.149 | 0.637±0.168 |
| KNN | 0.777±0.064 | 0.080±0.144 | 0.943±0.070 | 0.287±0.388 | 0.436±0.059 | 0.620±0.215 |
| Adaboost | 0.813±0.069 | 0.287±0.278 | 0.938±0.084 | 0.588±0.361 | 0.526±0.163 | 0.698±0.125 |

*Abbreviations:* AUC, area under the curve; LR, logistic regression; SVM, support vector machine; GBM, gradient boosting machine; NN, neural network; RF, random forest; KNN, k-nearest neighbors; AdaBoost, adaptive boosting.

**Supplementary Table 5.** Baseline characteristics compared between CMV reactivation and non-reactivation.

|  | **Non-reactivation** | **Reactivation** | **p.overall** |
| --- | --- | --- | --- |
|  | ***N=179*** | ***N=42*** |  |
| Age,years | 69.0 [60.0;77.0] | 66.0 [56.5;79.0] | 0.544 |
| Gender,male(%) | 123 (68.7%) | 32 (76.2%) | 0.444 |
| BMI | 22.8 (3.88) | 22.9 (4.39) | 0.938 |
| APACHEII | 20.0 [15.0;27.0] | 23.0 [18.0;30.0] | 0.037 |
| Infectious sites: |  |  | 0.940 |
| Lung | 113 (63.1%) | 26 (61.9%) |  |
| Abdomin | 44 (24.6%) | 10 (23.8%) |  |
| Bloodflow | 22 (12.3%) | 6 (14.3%) |  |
| DM,n(%) | 46 (25.7%) | 8 (19.0%) | 0.482 |
| CHD,n(%) | 22 (12.3%) | 3 (7.14%) | 0.428 |
| Organ transplantation,n(%) | 3 (1.68%) | 4 (9.52%) | 0.026 |
| CHF,n(%) | 12 (6.70%) | 5 (11.9%) | 0.330 |
| CKD,n(%) | 17 (9.50%) | 8 (19.0%) | 0.102 |
| ARDs,n(%) | 13 (7.26%) | 5 (11.9%) | 0.347 |
| Malignant tumor,n(%) | 66 (36.9%) | 11 (26.2%) | 0.260 |
| CMV IgG,AU/ml | 191 [147;250] | 250 [249;250] | <0.001 |
| Lymphocytes counts,x10^9/L | 0.80 [0.52;1.06] | 0.60 [0.38;0.99] | 0.058 |
| PLT,x10^9/L | 151 [93.8;230] | 146 [91.1;220] | 0.969 |
| Neu,x10^9/L | 9.20 [6.66;12.2] | 9.52 [7.10;13.4] | 0.396 |
| CRP,mg/L | 102 [59.2;166] | 90.7 [59.4;136] | 0.190 |
| ALT,IU/L | 26.2 [13.8;55.8] | 22.8 [14.5;40.5] | 0.711 |
| AST,IU/L | 38.2 [25.0;78.1] | 29.4 [23.0;52.5] | 0.222 |
| CD3^+^ lymphocyte count,/μl | 514 [304;744] | 396 [198;705] | 0.052 |
| CD4^+^ lymphocyte count,/μl | 312 [194;464] | 203 [98.5;329] | 0.003 |
| CD8^+^ lymphocyte count,/μl | 181 [98.5;274] | 167 [57.6;278] | 0.402 |
| APTT,s | 34.0 [30.2;39.2] | 34.5 [31.1;39.7] | 0.733 |
| PT,s | 13.7 [12.8;15.5] | 14.3 [12.8;16.9] | 0.526 |
| D-dimer,mg/L | 4.71 [2.59;8.56] | 4.02 [1.97;7.32] | 0.553 |
| PCT,ng/mL | 0.96 [0.30;7.10] | 0.96 [0.36;4.01] | 0.625 |
| Pre-alb,mg/L | 114 [76.2;136] | 119 [92.6;162] | 0.288 |
| Mechanical ventilation,days | 9.68 (10.0) | 12.2 (11.6) | 0.194 |
| Intravenous glucocorticoid,days | 0.00 [0.00;6.00] | 3.00 [0.00;13.2] | 0.003 |
| Vasoactive agents,days | 3.00 [0.00;8.00] | 6.50 [1.00;20.0] | 0.011 |
| 90-day mortality,n(%) | 42 (23.5%) | 18 (42.9%) | 0.019 |

Abbreviations: BMI, body mass index; APACHE II , acute physiology and chronic health evaluation II; DM, diabetes mellitus; CAHD, coronary atherosclerotic heart disease; CHF, chronic heart failure; CKD, chronic kidney disease; AIDs, autoimmune diseases; CMV, cytomegalovirus; ALT, alanine transaminase; AST, aspartate transaminase; APTT, activated partial thromboplastin time; PT, prothrombin time; PCT, procalcitonin.

**Supplementary Table 6.** Evaluation metrics of seven models derived from Training set processed with SMOTE.

| **Model** | **Threshold** | **Accuracy** | **Sensitivity** | **Specificity** | **Precision** | **F1 Score** | **AUC** |
| --- | --- | --- | --- | --- | --- | --- | --- |
| Traing |  |  |  |  |  |  |  |
| LR | 0.546 | 0.75(0.679–0.820) | 0.667(0.485–0.828) | 0.77(0.689–0.842) | 0.408(0.271–0.554) | 0.506(0.356–0.639) | 0.762 (0.661–0.862) |
| SVM | 0.592 | 0.769(0.699–0.833) | 0.667(0.484–0.829) | 0.794(0.718–0.862) | 0.435(0.286–0.583) | 0.526(0.378–0.658) | 0.760 (0.658–0.861) |
| GBM | 0.562 | 0.795(0.731–0.859) | 0.8(0.656–0.933) | 0.794(0.724–0.863) | 0.48(0.349–0.621) | 0.6(0.468–0.721) | 0.812 (0.720–0.905) |
| NN | 0.48 | 0.609(0.538–0.679) | 0.9(0.786–1.000) | 0.54(0.452–0.628) | 0.318(0.225–0.417) | 0.47(0.353–0.574) | 0.731 (0.641–0.822) |
| RF | 0.995 | 1(-) | 1(-) | 1(-) | 1(-) | 1(-) | 1.000 (–) |
| KNN | 0.5 | 0.846(0.788–0.897) | 1(-) | 0.81(0.739–0.872) | 0.556(0.434–0.680) | 0.714(0.605–0.810) | 0.905 (0.870–0.939) |
| AdaBoost | 0.5 | 0.712(0.641–0.782) | 0.8(0.650–0.933) | 0.69(0.612–0.772) | 0.381(0.263–0.500) | 0.516(0.383–0.632) | 0.789 (0.695–0.884) |
| Test |  |  |  |  |  |  |  |
| LR | 0.488 | 0.692(0.569–0.800) | 0.833(0.583–1.000) | 0.66(0.526–0.778) | 0.357(0.174–0.529) | 0.5(0.279–0.678) | 0.668 (0.501–0.835) |
| SVM | 0.465 | 0.631(0.508–0.738) | 0.833(0.583–1.000) | 0.585(0.453–0.709) | 0.312(0.150–0.471) | 0.455(0.250–0.622) | 0.646 (0.470–0.822) |
| GBM | 0.475 | 0.738(0.631–0.846) | 0.667(0.385–0.929) | 0.755(0.640–0.870) | 0.381(0.176–0.611) | 0.485(0.250–0.667) | 0.722 (0.547–0.896) |
| NN | 0.524 | 0.662(0.538–0.769) | 0.75(0.455–1.000) | 0.642(0.510–0.765) | 0.321(0.143–0.500) | 0.45(0.222–0.629) | 0.691 (0.515–0.867) |
| RF | 0.627 | 0.769(0.662–0.862) | 0.583(0.267–0.889) | 0.811(0.700–0.911) | 0.412(0.176–0.647) | 0.483(0.222–0.690) | 0.625 (0.428–0.822) |
| KNN | 0.5 | 0.677(0.554–0.785) | 0.583(0.267–0.889) | 0.698(0.564–0.820) | 0.304(0.118–0.480) | 0.4(0.174–0.579) | 0.641 (0.482–0.799) |
| AdaBoost | 0.5 | 0.723(0.615–0.831) | 0.667(0.385–0.929) | 0.736(0.615–0.844) | 0.364(0.158–0.562) | 0.471(0.240–0.667) | 0.676 (0.511–0.841) |

*Abbreviations:* AUC, area under the curve; LR, logistic regression; SVM, support vector machine; GBM, gradient boosting machine; NN, neural network; RF, random forest; KNN, k-nearest neighbors; AdaBoost, adaptive boosting.
