# Supplementary figures and images for: Risk stratification for CMV reactivation in sepsis patients: development of an interpretable machine learning model
Source: BMC Infect Dis. 2025 Dec 22;25:1729. doi: 10.1186/s12879-025-12154-0 (PMC12723881; doi:10.1186/s12879-025-12154-0)

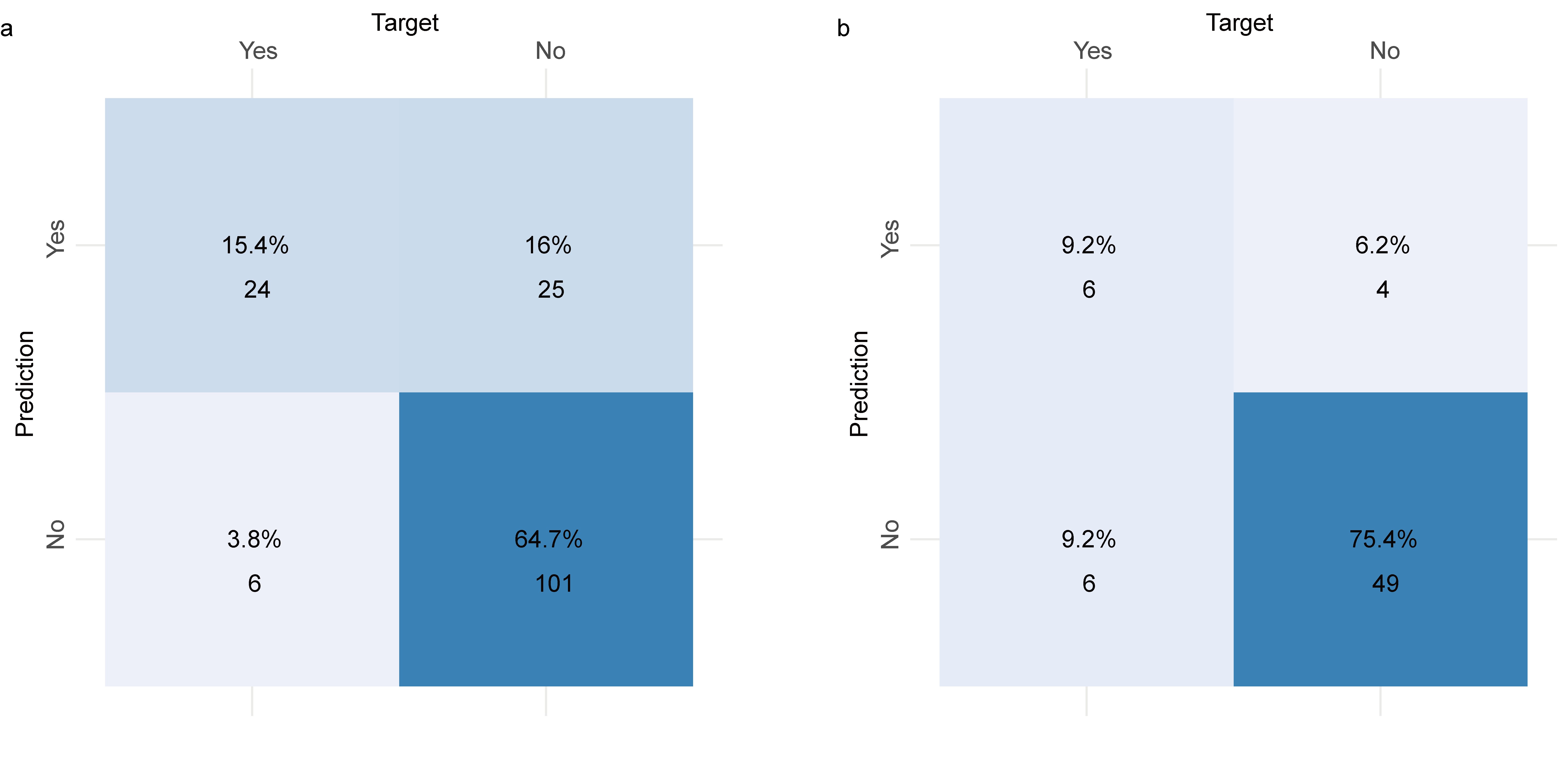

Supplement: Supplementary file 2 — Supplementary Material 2 [file 12879_2025_12154_MOESM2_ESM.jpg]

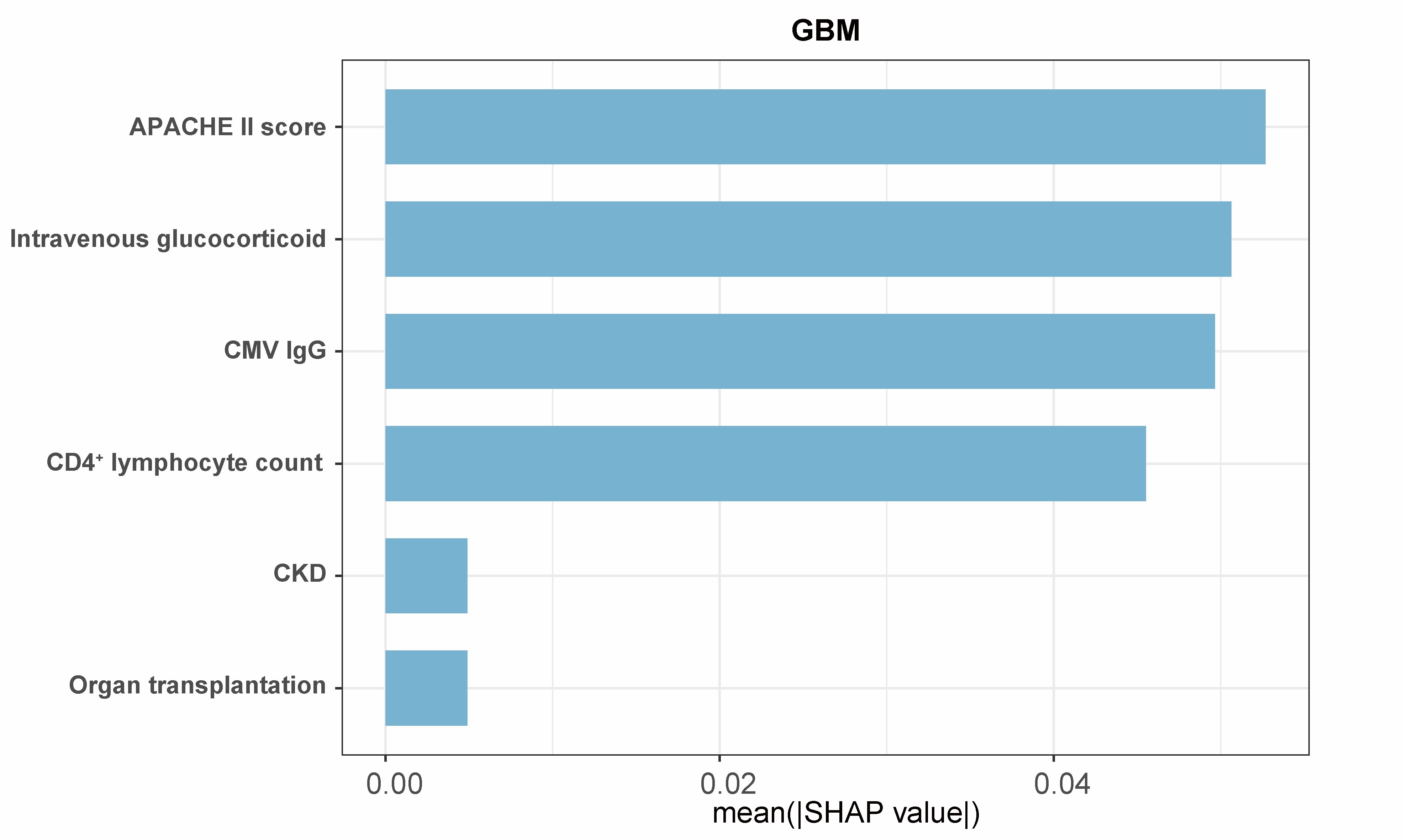

Supplement: Supplementary file 3 — Supplementary Material 3 [file 12879_2025_12154_MOESM3_ESM.jpg]

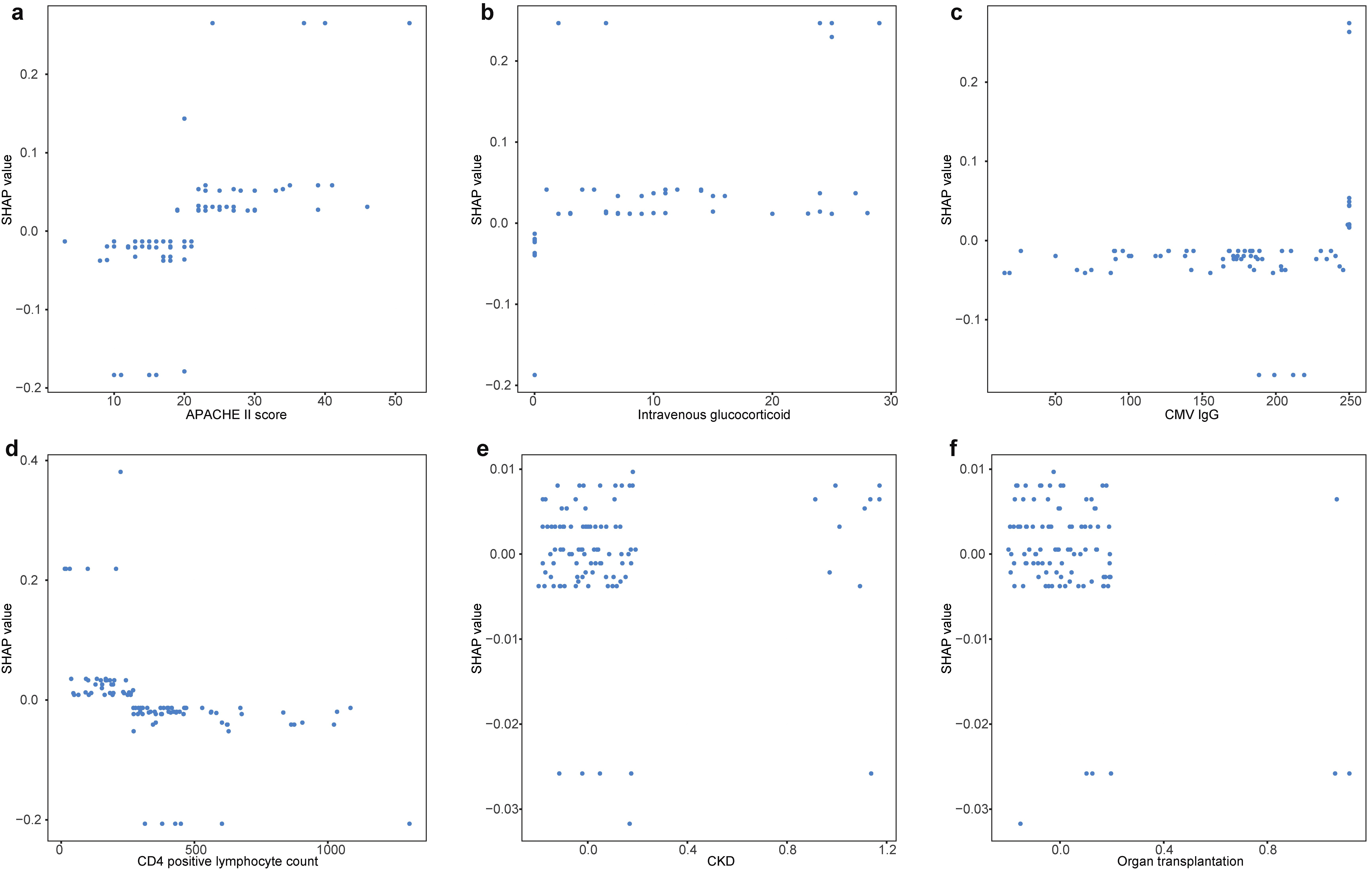

Supplement: Supplementary file 4 — Supplementary Material 4 [file 12879_2025_12154_MOESM4_ESM.jpg]

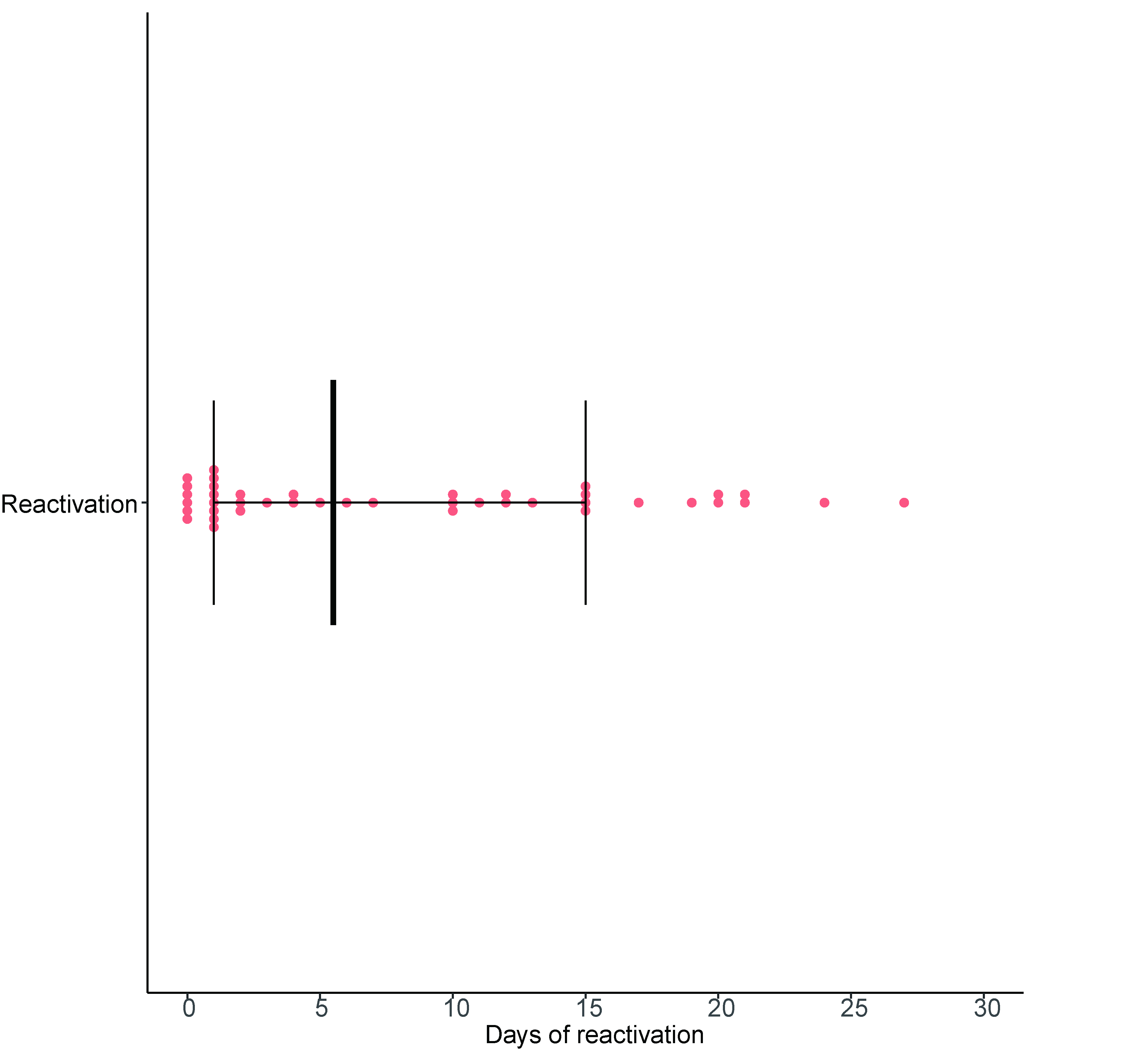

Supplement: Supplementary file 5 — Supplementary Material 5 [file 12879_2025_12154_MOESM5_ESM.jpg]
