# Supplementary material for: Risk stratification for CMV reactivation in sepsis patients: development of an interpretable machine learning model
Source: BMC Infect Dis. 2025 Dec 22;25:1729. doi: 10.1186/s12879-025-12154-0 (PMC12723881; doi:10.1186/s12879-025-12154-0)
Supplement: Supplementary file 6 — Supplementary Material 6 [file 12879_2025_12154_MOESM6_ESM.docx]

**Supplementary Table 1.** Sequential Organ Failure Assessment Score

| **System** | **Score** | | | | |
| --- | --- | --- | --- | --- | --- |
|  | **0** | **1** | **2** | **3** | **4** |
| PaO_2_/FIO_2_, mm Hg   (kPa) | ≥400 (53.3) | <400 (53.3) | <300 (40) | <200 (26.7) with respiratory support | <100 (13.3) with respiratory support |
| Platelets, ×10^3^/µL | ≥150 | <150 | <100 | <50 | <20 |
| Bilirubin, mg/dL   (µmol/L) | <1.2 (20) | 1.2–1.9 (20–32) | 2.0–5.9 (33–101) | 6.0–11.9 (102–204) | >12.0 (204) |
| Cardiovascular  (µg/kg/min) | MAP ≥70 mm Hg | MAP <70 mm Hg | Dopamine <5 or dobutamine (any dose) | Dopamine 5.1–15 or epinephrine ≤0.1 or norepinephrine ≤0.1 | Dopamine >15 or epinephrine >0.1 or norepinephrine >0.1 |
| Glasgow Coma Scale score | 15 | 13–14 | 10–12 | 6–9 | <6 |
| Creatinine, mg/dL   (µmol/L) | <1.2 (110) | 1.2–1.9 (110–170) | 2.0–3.4 (171–299) | 3.5–4.9 (300–440) | >5.0 (440) |
| Urine output, mL/d |  |  |  | <500 | <200 |

*Abbreviations*: FIO_2_, fraction of inspired oxygen; MAP, mean arterial pressure; PaO_2_, partial pressure of oxygen.
