# Supplementary material for: Risk stratification for CMV reactivation in sepsis patients: development of an interpretable machine learning model
Source: BMC Infect Dis. 2025 Dec 22;25:1729. doi: 10.1186/s12879-025-12154-0 (PMC12723881; doi:10.1186/s12879-025-12154-0)
Supplement: Supplementary file 7 — Supplementary Material 7 [file 12879_2025_12154_MOESM7_ESM.docx]

**Supplementary Table 2.** Key hyperparameters used in models

| **Model** | **Hyperparameter** | **Value(s)** |
| --- | --- | --- |
| LR | Not applied | Not applied |
| SVM | sigma | 0.001 |
|  | C | 0.09 |
| GBM | n.trees | 100 |
|  | interaction.depth | 5 |
|  | shrinkage | 0.1 |
|  | n.minobsinnode | 30 |
| NN | size | 6 |
|  | decay | 0.6 |
| RF | mtry | 11 |
|  | numRandomCuts | 3 |
| KNN | kmax | 12 |
|  | distance | 1 |
|  | kernel | optimal |
| AdaBoost | mfinal | 2 |
|  | maxdepth | 2 |
|  | coeflearn | Zhu |

*Abbreviations:* LR, logistic regression; SVM, support vector machine; GBM, gradient boosting machine; NN, neural network; RF, random forest; KNN, k-nearest neighbors; AdaBoost, adaptive boosting.
