# Supplementary material for: Risk stratification for CMV reactivation in sepsis patients: development of an interpretable machine learning model
Source: BMC Infect Dis. 2025 Dec 22;25:1729. doi: 10.1186/s12879-025-12154-0 (PMC12723881; doi:10.1186/s12879-025-12154-0)
Supplement: Supplementary file 8 — Supplementary Material 8 [file 12879_2025_12154_MOESM8_ESM.docx]

**Supplementary Table 3.** Mainly R packages with versions used.

| **Package** | **Version** |
| --- | --- |
| caret | 7.0-1 |
| e1071 | 1.7-16 |
| gbm | 2.2.2 |
| nnet | 7.3-19 |
| extraTrees | 1.0.5 |
| kknn | 1.3.1 |
| adabag | 5.0 |
| stats | 4.3.3 |
| pROC | 1.18.5 |
| ROCR | 1.0-11 |
| boot | 1.3-31 |
| Metrics | 0.1.4 |
| DALEX | 2.4.3 |
| kernelshap | 0.5.0 |
| shapviz | 0.9.6 |
| rmda | 1.6 |
| dcurves | 0.5.0 |
| ResourceSelection | 0.3-6 |
| DynNom | 5.1 |
| ROSE | 0.0-4 |
| DMwR | 0.4.1 |
| compareGroups | 4.9.1 |
