# Supplementary material for: Risk stratification for CMV reactivation in sepsis patients: development of an interpretable machine learning model
Source: BMC Infect Dis. 2025 Dec 22;25:1729. doi: 10.1186/s12879-025-12154-0 (PMC12723881; doi:10.1186/s12879-025-12154-0)
Supplement: Supplementary file 9 — Supplementary Material 9 [file 12879_2025_12154_MOESM9_ESM.docx]

**Supplementary Table 4.** 10-Fold Cross-Validated Performance Metrics with Mean ± Standard Deviation for Training Set

| **Model** | **Accuracy** | **Sensitivity** | **Specificity** | **Precision** | **F1 Score** | **AUC** |
| --- | --- | --- | --- | --- | --- | --- |
| LR | 0.801±0.060 | 0.140±0.166 | 0.959±0.068 | 0.535±0.454 | 0.447±0.083 | 0.727±0.156 |
| SVM | 0.802±0.018 | 0.027±0.091 | 0.987±0.030 | 0.250±0.267 | 0.400±0.000 | 0.619±0.202 |
| GBM | 0.818±0.062 | 0.193±0.203 | 0.967±0.051 | 0.624±0.428 | 0.501±0.121 | 0.759±0.157 |
| NN | 0.799±0.055 | 0.140±0.214 | 0.957±0.063 | 0.447±0.387 | 0.479±0.136 | 0.615±0.205 |
| RF | 0.769±0.086 | 0.280±0.246 | 0.886±0.094 | 0.407±0.332 | 0.447±0.149 | 0.637±0.168 |
| KNN | 0.777±0.064 | 0.080±0.144 | 0.943±0.070 | 0.287±0.388 | 0.436±0.059 | 0.620±0.215 |
| Adaboost | 0.813±0.069 | 0.287±0.278 | 0.938±0.084 | 0.588±0.361 | 0.526±0.163 | 0.698±0.125 |

*Abbreviations:* AUC, area under the curve; LR, logistic regression; SVM, support vector machine; GBM, gradient boosting machine; NN, neural network; RF, random forest; KNN, k-nearest neighbors; AdaBoost, adaptive boosting.
