# Supplementary material for: Risk stratification for CMV reactivation in sepsis patients: development of an interpretable machine learning model
Source: BMC Infect Dis. 2025 Dec 22;25:1729. doi: 10.1186/s12879-025-12154-0 (PMC12723881; doi:10.1186/s12879-025-12154-0)
Supplement: Supplementary file 10 — Supplementary Material 10 [file 12879_2025_12154_MOESM10_ESM.docx]

**Supplementary Table 5.** Baseline characteristics compared between CMV reactivation and non-reactivation.

|  | **Non-reactivation** | **Reactivation** | **p.overall** |
| --- | --- | --- | --- |
|  | ***N=179*** | ***N=42*** |  |
| Age,years | 69.0 [60.0;77.0] | 66.0 [56.5;79.0] | 0.544 |
| Gender,male(%) | 123 (68.7%) | 32 (76.2%) | 0.444 |
| BMI | 22.8 (3.88) | 22.9 (4.39) | 0.938 |
| APACHEII | 20.0 [15.0;27.0] | 23.0 [18.0;30.0] | 0.037 |
| Infectious sites: |  |  | 0.940 |
| Lung | 113 (63.1%) | 26 (61.9%) |  |
| Abdomin | 44 (24.6%) | 10 (23.8%) |  |
| Bloodflow | 22 (12.3%) | 6 (14.3%) |  |
| DM,n(%) | 46 (25.7%) | 8 (19.0%) | 0.482 |
| CHD,n(%) | 22 (12.3%) | 3 (7.14%) | 0.428 |
| Organ transplantation,n(%) | 3 (1.68%) | 4 (9.52%) | 0.026 |
| CHF,n(%) | 12 (6.70%) | 5 (11.9%) | 0.330 |
| CKD,n(%) | 17 (9.50%) | 8 (19.0%) | 0.102 |
| ARDs,n(%) | 13 (7.26%) | 5 (11.9%) | 0.347 |
| Malignant tumor,n(%) | 66 (36.9%) | 11 (26.2%) | 0.260 |
| CMV IgG,AU/ml | 191 [147;250] | 250 [249;250] | <0.001 |
| Lymphocytes counts,x10^9/L | 0.80 [0.52;1.06] | 0.60 [0.38;0.99] | 0.058 |
| PLT,x10^9/L | 151 [93.8;230] | 146 [91.1;220] | 0.969 |
| Neu,x10^9/L | 9.20 [6.66;12.2] | 9.52 [7.10;13.4] | 0.396 |
| CRP,mg/L | 102 [59.2;166] | 90.7 [59.4;136] | 0.190 |
| ALT,IU/L | 26.2 [13.8;55.8] | 22.8 [14.5;40.5] | 0.711 |
| AST,IU/L | 38.2 [25.0;78.1] | 29.4 [23.0;52.5] | 0.222 |
| CD3^+^ lymphocyte count,/μl | 514 [304;744] | 396 [198;705] | 0.052 |
| CD4^+^ lymphocyte count,/μl | 312 [194;464] | 203 [98.5;329] | 0.003 |
| CD8^+^ lymphocyte count,/μl | 181 [98.5;274] | 167 [57.6;278] | 0.402 |
| APTT,s | 34.0 [30.2;39.2] | 34.5 [31.1;39.7] | 0.733 |
| PT,s | 13.7 [12.8;15.5] | 14.3 [12.8;16.9] | 0.526 |
| D-dimer,mg/L | 4.71 [2.59;8.56] | 4.02 [1.97;7.32] | 0.553 |
| PCT,ng/mL | 0.96 [0.30;7.10] | 0.96 [0.36;4.01] | 0.625 |
| Pre-alb,mg/L | 114 [76.2;136] | 119 [92.6;162] | 0.288 |
| Mechanical ventilation,days | 9.68 (10.0) | 12.2 (11.6) | 0.194 |
| Intravenous glucocorticoid,days | 0.00 [0.00;6.00] | 3.00 [0.00;13.2] | 0.003 |
| Vasoactive agents,days | 3.00 [0.00;8.00] | 6.50 [1.00;20.0] | 0.011 |
| 90-day mortality,n(%) | 42 (23.5%) | 18 (42.9%) | 0.019 |

Abbreviations: BMI, body mass index; APACHE II , acute physiology and chronic health evaluation II; DM, diabetes mellitus; CAHD, coronary atherosclerotic heart disease; CHF, chronic heart failure; CKD, chronic kidney disease; AIDs, autoimmune diseases; CMV, cytomegalovirus; ALT, alanine transaminase; AST, aspartate transaminase; APTT, activated partial thromboplastin time; PT, prothrombin time; PCT, procalcitonin.
