# Supplementary material for: Risk stratification for CMV reactivation in sepsis patients: development of an interpretable machine learning model
Source: BMC Infect Dis. 2025 Dec 22;25:1729. doi: 10.1186/s12879-025-12154-0 (PMC12723881; doi:10.1186/s12879-025-12154-0)
Supplement: Supplementary file 11 — Supplementary Material 11 [file 12879_2025_12154_MOESM11_ESM.docx]

**Supplementary Table 6.** Evaluation metrics of seven models derived from Training set processed with SMOTE.

| **Model** | **Threshold** | **Accuracy** | **Sensitivity** | **Specificity** | **Precision** | **F1 Score** | **AUC** |
| --- | --- | --- | --- | --- | --- | --- | --- |
| Traing |  |  |  |  |  |  |  |
| LR | 0.546 | 0.75(0.679–0.820) | 0.667(0.485–0.828) | 0.77(0.689–0.842) | 0.408(0.271–0.554) | 0.506(0.356–0.639) | 0.762 (0.661–0.862) |
| SVM | 0.592 | 0.769(0.699–0.833) | 0.667(0.484–0.829) | 0.794(0.718–0.862) | 0.435(0.286–0.583) | 0.526(0.378–0.658) | 0.760 (0.658–0.861) |
| GBM | 0.562 | 0.795(0.731–0.859) | 0.8(0.656–0.933) | 0.794(0.724–0.863) | 0.48(0.349–0.621) | 0.6(0.468–0.721) | 0.812 (0.720–0.905) |
| NN | 0.48 | 0.609(0.538–0.679) | 0.9(0.786–1.000) | 0.54(0.452–0.628) | 0.318(0.225–0.417) | 0.47(0.353–0.574) | 0.731 (0.641–0.822) |
| RF | 0.995 | 1(-) | 1(-) | 1(-) | 1(-) | 1(-) | 1.000 (–) |
| KNN | 0.5 | 0.846(0.788–0.897) | 1(-) | 0.81(0.739–0.872) | 0.556(0.434–0.680) | 0.714(0.605–0.810) | 0.905 (0.870–0.939) |
| AdaBoost | 0.5 | 0.712(0.641–0.782) | 0.8(0.650–0.933) | 0.69(0.612–0.772) | 0.381(0.263–0.500) | 0.516(0.383–0.632) | 0.789 (0.695–0.884) |
| Test |  |  |  |  |  |  |  |
| LR | 0.488 | 0.692(0.569–0.800) | 0.833(0.583–1.000) | 0.66(0.526–0.778) | 0.357(0.174–0.529) | 0.5(0.279–0.678) | 0.668 (0.501–0.835) |
| SVM | 0.465 | 0.631(0.508–0.738) | 0.833(0.583–1.000) | 0.585(0.453–0.709) | 0.312(0.150–0.471) | 0.455(0.250–0.622) | 0.646 (0.470–0.822) |
| GBM | 0.475 | 0.738(0.631–0.846) | 0.667(0.385–0.929) | 0.755(0.640–0.870) | 0.381(0.176–0.611) | 0.485(0.250–0.667) | 0.722 (0.547–0.896) |
| NN | 0.524 | 0.662(0.538–0.769) | 0.75(0.455–1.000) | 0.642(0.510–0.765) | 0.321(0.143–0.500) | 0.45(0.222–0.629) | 0.691 (0.515–0.867) |
| RF | 0.627 | 0.769(0.662–0.862) | 0.583(0.267–0.889) | 0.811(0.700–0.911) | 0.412(0.176–0.647) | 0.483(0.222–0.690) | 0.625 (0.428–0.822) |
| KNN | 0.5 | 0.677(0.554–0.785) | 0.583(0.267–0.889) | 0.698(0.564–0.820) | 0.304(0.118–0.480) | 0.4(0.174–0.579) | 0.641 (0.482–0.799) |
| AdaBoost | 0.5 | 0.723(0.615–0.831) | 0.667(0.385–0.929) | 0.736(0.615–0.844) | 0.364(0.158–0.562) | 0.471(0.240–0.667) | 0.676 (0.511–0.841) |

*Abbreviations:* AUC, area under the curve; LR, logistic regression; SVM, support vector machine; GBM, gradient boosting machine; NN, neural network; RF, random forest; KNN, k-nearest neighbors; AdaBoost, adaptive boosting.
